# Lactate- and immunomagnetic-purified hiPSC–derived cardiomyocytes generate comparable engineered cardiac tissue constructs

**DOI:** 10.1172/jci.insight.172168

**Published:** 2024-01-09

**Authors:** Kalina J. Rossler, Willem J. de Lange, Morgan W. Mann, Timothy J. Aballo, Jake A. Melby, Jianhua Zhang, Gina Kim, Elizabeth F. Bayne, Yanlong Zhu, Emily T. Farrell, Timothy J. Kamp, J. Carter Ralphe, Ying Ge

**Affiliations:** 1 Molecular and Cellular Pharmacology Training Program,; 2 Department of Cell and Regenerative Biology,; 3 Department of Pediatrics,; 4 Department of Medicine,; 5 Department of Chemistry, and; 6 Human Proteomics Program, School of Medicine and Public Health, University of Wisconsin-Madison, Madison, Wisconsin, USA.

**Keywords:** Cardiology, Stem cells, Cardiovascular disease, iPS cells

## Abstract

Three-dimensional engineered cardiac tissue (ECT) using purified human induced pluripotent stem cell–derived cardiomyocytes (hiPSC-CMs) has emerged as an appealing model system for the study of human cardiac biology and disease. A recent study reported widely used metabolic (lactate) purification of monolayer hiPSC-CM cultures results in an ischemic cardiomyopathy-like phenotype compared with magnetic antibody-based cell sorting (MACS) purification, complicating the interpretation of studies using lactate-purified hiPSC-CMs. Herein, our objective was to determine if use of lactate relative to MACS-purified hiPSC-CMs affects the properties of resulting hiPSC-ECTs. Therefore, hiPSC-CMs were differentiated and purified using either lactate-based media or MACS. Global proteomics revealed that lactate-purified hiPSC-CMs displayed a differential phenotype over MACS hiPSC-CMs. hiPSC-CMs were then integrated into 3D hiPSC-ECTs and cultured for 4 weeks. Structurally, there was no significant difference in sarcomere length between lactate and MACS hiPSC-ECTs. Assessment of isometric twitch force and Ca^2+^ transient measurements revealed similar functional performance between purification methods. High-resolution mass spectrometry–based quantitative proteomics showed no significant difference in protein pathway expression or myofilament proteoforms. Taken together, this study demonstrates that lactate- and MACS-purified hiPSC-CMs generate ECTs with comparable structural, functional, and proteomic features, and it suggests that lactate purification does not result in an irreversible change in a hiPSC-CM phenotype.

## Abstract 2

<p>Lactate-purified human-induced pluripotent stem cell&ndash;derived cardiomyocytes are functionally, structurally, and phenotypically comparable to those purified via magnetic antibody-based cell sorting.</p>

## Graphical abstract

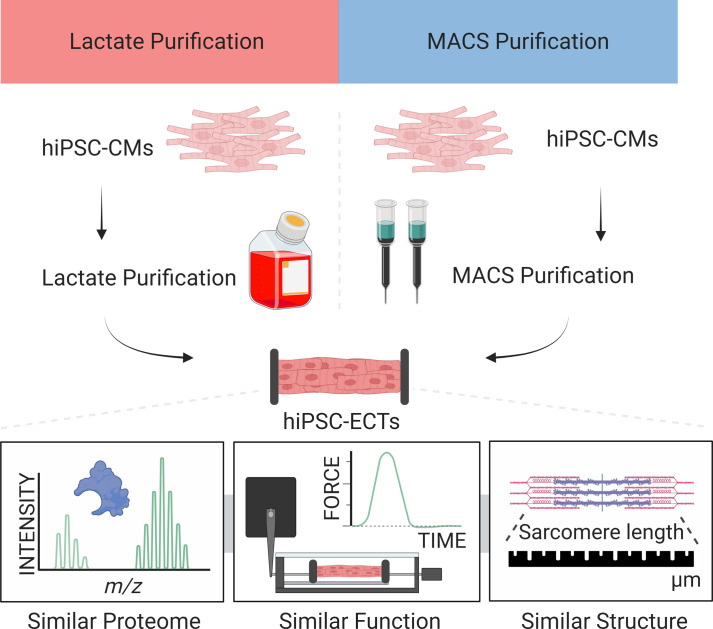

## Introduction

Human induced pluripotent stem cell–derived cardiomyocytes (hiPSC-CMs) have emerged as an important in vitro tool for cardiovascular disease modeling, cardiotoxicity screening, and drug discovery (1, 2). Both monogenic and acquired cardiac diseases have been modeled using 2-dimensional (2D) hiPSC-CMs (3–6). While hiPSC-CMs permit biological studies in a fully human context, the relative immaturity of hiPSC-CMs remains a notable limitation when considering their application (7, 8). Considerable efforts have been devoted to improving the developmental and maturation state of hiPSC-CM models, including, but not limited to, the implementation of extending time in culture, treating with specific growth factors, applying mechanical and electrical stimulation, coculturing with other cell types, and seeding cells in 3-dimensional (3D) matrices (9–11). Recent studies showed coculturing purified hiPSC-CMs with cardiac fibroblasts (CFs) within 3D engineered cardiac tissue (hiPSC-ECT) constructs improved maturation and function relative to 2D monolayer cultures. The hiPSC-ECT showed evidence of t-tubule systems, more mature intracellular calcium handling, and protein isoform switching — all of which are consistent with more mature heart tissue (12, 13). Thus, hiPSC-ECTs have great potential for enhanced in vitro cardiac modeling.

The first step in hiPSC-ECT generation is the creation of a pure hiPSC-CM population. Purification after hiPSC-CM differentiation is necessary to ensure a more uniform final cell composition within the modeling matrix (14–16). This is achieved either by immunomagnetic means — labeling cells using a biotin conjugate and passing through a microbead column (magnetic-activated cell sorting [MACS]) — or metabolic purification using a lactate-based media (17). To date, the most popular and cost-effective method for the purification of 2D hiPSC-CMs is the use of glucose-free, lactate media. This approach relies on the unique ability of CMs to effectively utilize lactate as a metabolic substrate over other cells (18). Thus, lactate purification is currently preferred by many laboratories for generation of 2D and 3D hiPSC-CM models (15, 19–39). Notably, lactate-purified hiPSC-CMs have been used to test new therapeutic agents for diseases, such as Duchenne muscular dystrophy (34) and Lamin induced cardiomyopathies (40).

Despite its widespread use, questions regarding the metabolic stress caused by lactate selection have been raised. A recent study by Davis et al. suggests lactate purification of 2D hiPSC-CM cultures results in an “ischemic-like” phenotype modeling ischemic heart failure in vitro, making them less desirable for widespread applications (3). The lactate-purified hiPSC-CMs exhibited changes in molecular composition, structure, and function relative to MACS-purified hiPSC-CMs. For example, lactate-purified hiPSC-CMs displayed spontaneous arrhythmic activity, abnormal calcium handling, shortened sarcomeres, and aberrant contractile protein expression. Based on the concerns raised by this work, our objective was to investigate if the responses to lactate purification identified in Davis et al. in 2D-cultured hiPSC-CMs represented a transient metabolic adaptation or a persistent phenotypic change. We hypothesized that prolonged culture of lactate- or MACS-purified hiPSC-CMs in a 3D environment in hiPSC-ECTs for 4 weeks would generate comparable functioning cardiac tissue.

First, we compared 2D hiPSC-CM monolayer cultures directly after lactate and MACS purification using global proteomics and confirmed a distinct phenotype in our lactate-purified hiPSC-CMs. Next, we compared the structure, function, and protein expression of hiPSC-ECTs containing either lactate-purified hiPSC-CMs or hiPSC-CMs purified using a commercially available MACS kit (Miltenyi Biotec). Notably, once incorporated in hiPSC-ECTs, we observed no differences in overall structure and performed IHC staining of the cardiac *Z* discs to quantify sarcomere length. We assessed the functional performance and calcium transients (Ca^2+^TR) of our hiPSC-ECTs. Moreover, we utilized an unbiased high-resolution mass spectrometry–based (MS-based) proteomics method that integrated both bottom-up global proteomics, to comprehensively assess global proteome changes, and top-down targeted proteomics, to accurately determine the changes in sarcomeric posttranslational modifications (PTMs) and isoforms, collectively called proteoforms (13, 41). This detailed analysis of structure, function, and proteome revealed no substantial differences between lactate and MACS hiPSC-ECTs. Taken together, this study suggests that lactate or MACS-purified hiPSC-CMs can be comparably used in 3D culture platforms, resulting in similar structural, functional, and molecular characteristics of mature hiPSC-ECTs.

## Results

### Comparison of lactate and MACS-purified hiPSC-CM.

**Figure 1:**
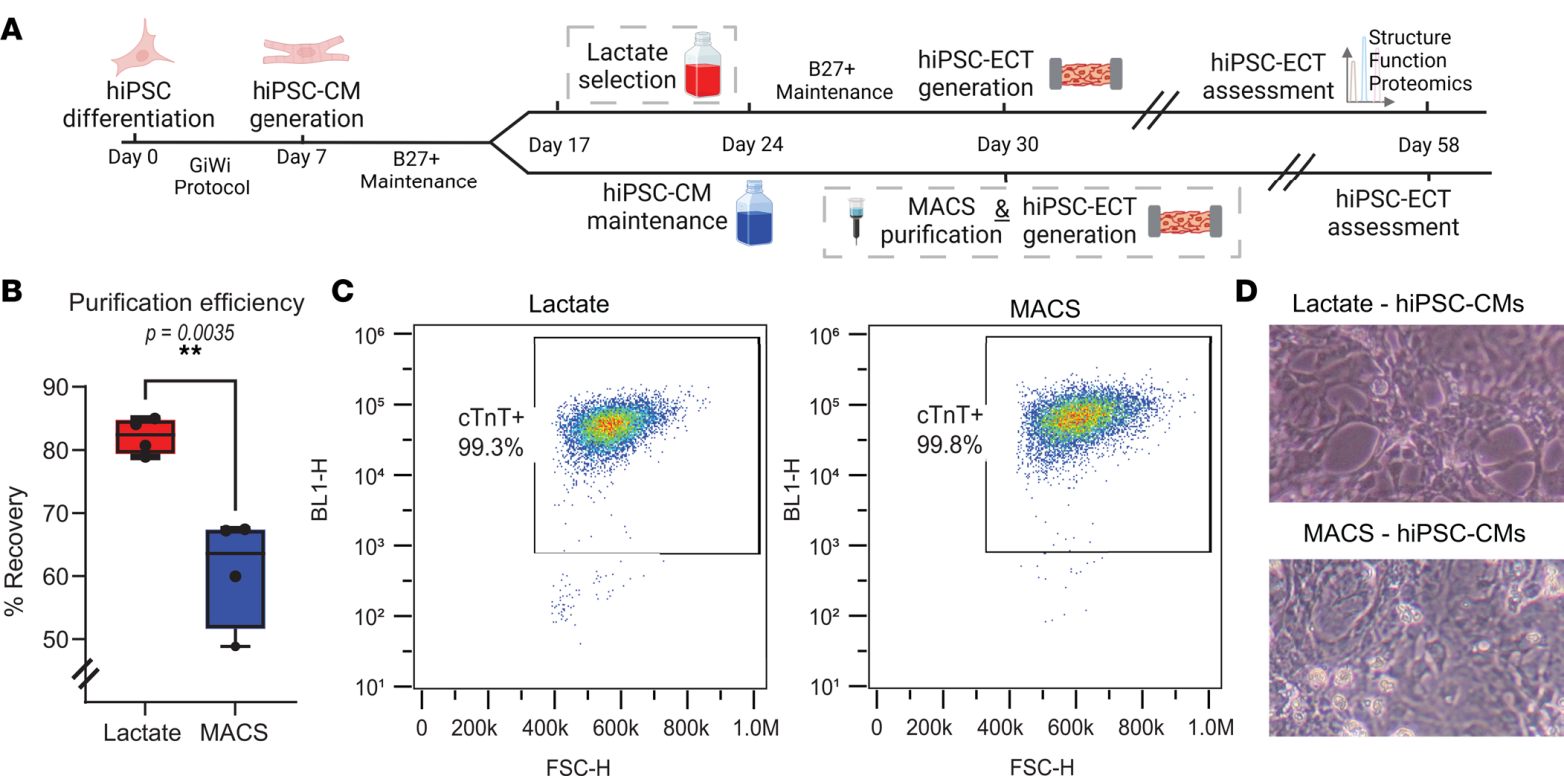
Purification of 2D hiPSC-CMs for 3D hiPSC-ECT generation. (**A**) Timeline for the generation of hiPSC-CMs and hiPSC-ECTs. (**B**) Efficiency of purification methods are given by percentage of pure hiPSC-CMs recovered from successful differentiation batches. Viable differentiation batches were visually evaluated as viable with observance of greater than 80% of cells contracting in the well. Percentage recovery was calculated as follows: Percentage recovery = (Count of cTnT^+^ cells after purification process/Count of total cells before purification process) × 100. Lactate differentiation was significantly more successful in pure hiPSC-CM recovery in comparison with MACS (lactate = 82.2% ± 2.86 %, versus MACS = 60.9% ± 8.73 %; *P* = 0.0035). All tests were performed with 4 separate differentiation batches split to purification process. Lactate purification occurred from hiPSC-CM day 17 to day 24, with day 0 as the start of hiPSC differentiation. MACS purification occurred at day 30 before generation of hiPSC-ECTs. Statistical analysis involved 2-tailed Student’s *t* test with α = 0.05. (**C**) Flow cytometry with cTnT labeling demonstrates effective hiPSC-CM enrichment using each purification method. Representative experiment performed once. (**D**) Two-dimensional representations of hiPSC-CMs from lactate and MACS purification methods. Two-dimensional hiPSC-CM images were taken on the day of hiPSC-ECT generation with 400× Olympus microscope. Representative experiment performed once.

We utilized an established control hiPSC line (Stanford University Cardiovascular Institutes Biobank, Stanford, California, USA; Hypertrophic Cardiomyopathy/*MYH7*/Control iPSC line) and a monolayer-based, biphasic modulation of Wnt signaling protocol (GiWi protocol) (4, 42) to generate hiPSC-CMs and hiPSC-ECTs for our study (Figure 1A and Supplemental Data WiCell Report; supplemental material available online with this article; https://doi.org/10.1172/jci.insight.172168DS1). We then enriched our differentiated hiPSC-CM cultures using either a lactate-based media or magnetic antibody-based (MACS) depletion of the nonmyocyte cell populations. Each differentiation batch of hiPSC-CMs was applied to both arms (lactate versus MACS) of our study to ensure well-matched comparisons. Recovery of pure hiPSC-CMs after purification was greater using lactate purification versus MACS (Figure 1B; lactate = 82.2% ± 2.86 % [*n* = 4] versus MACS = 60.9% ± 8.73 % [*n* = 4]; *P* = 0.0035). Notably, hiPSC-CM purification using both methods yielded similar percentages of pure CMs (Figure 1C) as previously described (3). No obvious morphological differences were observed in 2D cells between lactate and MACS purification methods (Figure 1D).

We sought to determine if lactate purification altered the phenotype of the iPSC-CMs relative to MACS selection, as observed by Davis et al. (3) in our 2D hiPSC-CMs. To provide an unbiased assessment of these cell populations, we employed global expression proteomic profiling. Lactate purification occurred from day 17 to day 24, and MACS purification was performed on day 24. On day 24, hiPSC-CMs were harvested from 2D monolayers, and a buffer containing photocleavable surfactant, 4-hexylphenylazosulfonate (Azo) (0.25% w/v), was used to extract the global proteome similar to established protocols (43, 44).

**Figure 2:**
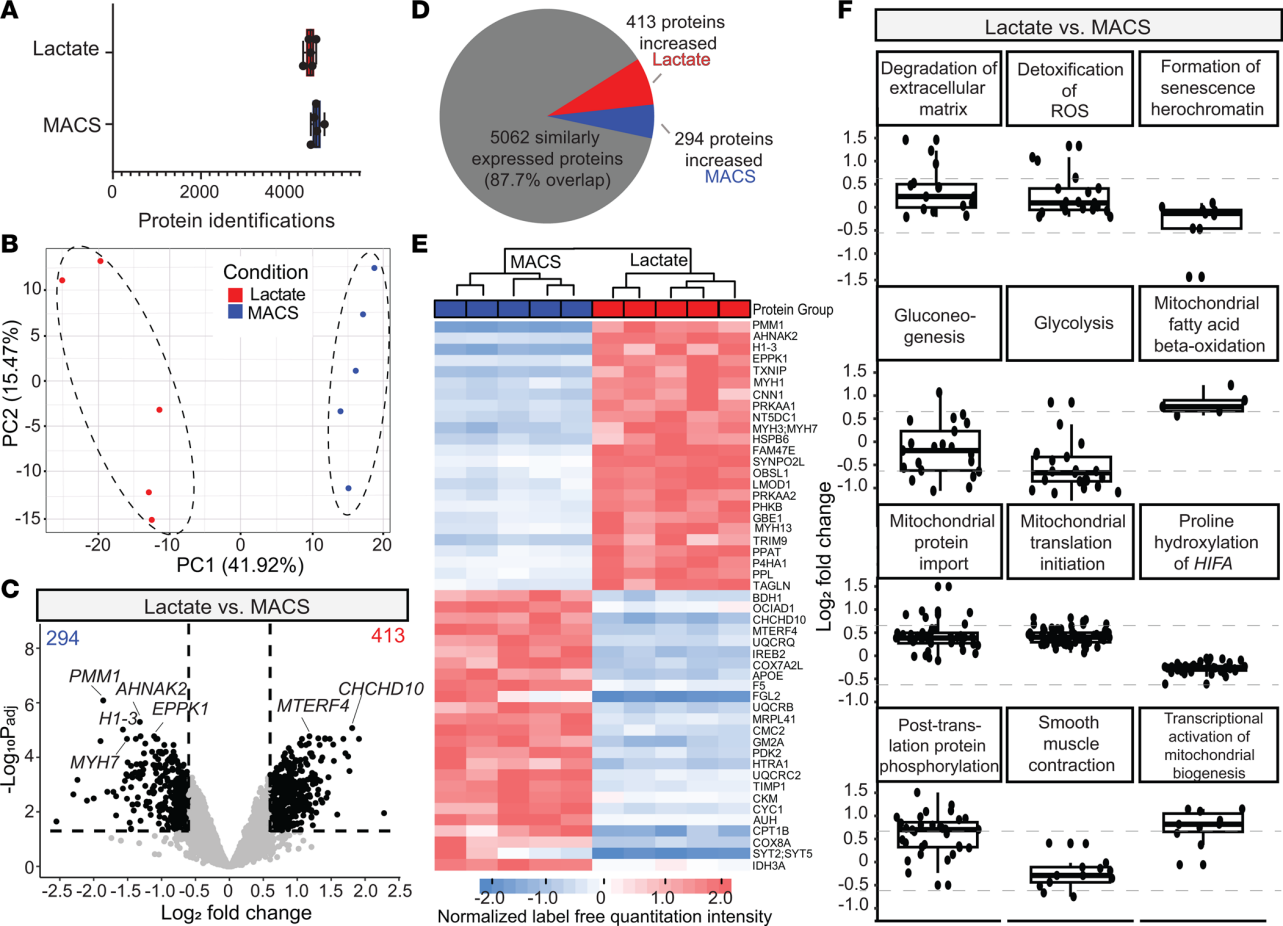
Global proteomics analysis of hiPSC-CMs after purification. (**A**) Unique protein identifications per hiPSC-ECT group. (**B**) Principal component analysis (PCA) of sample log_2_ protein abundances illustrates clear separation between purification techniques. (**C**) Volcano plot illustrating differential fold-change in protein expression between lactate and MACS purification. The number of significantly upregulated proteins is shown in the top outside corners of the plot (*P*_adj_ ≤ 0.05 and |log_2_ fold change| ≥ 0.6 required to be considered significant). Some significant proteins are indicated by name on the plot. (**D**) Pie chart for visual representation of differentially expressed proteins. (**E**) Hierarchal unbiased dendrogram clustering and heatmap depicting normalized intensities of *Z* scores for the top 50 significantly differently expressed proteins between lactate and MACS purification. (**F**) UniProt/Reactome pathways with pathways including keywords “muscle”, “cardiomyopathy”, “reactive oxygen species”, “glycolysis”, “beta-oxidation”, “microtubule”, “hypoxia”, “senescence”, “TGFβ”, “adrenaline”, “trafficking”, “hypertrophy,” and “mitochondrial”. Points indicate individual proteins identified in the pathway, and the box plots indicate average expression change of those proteins. Significantly differentially expressed proteins and pathways are indicated by log_2_ fold change greater than 0.6 or less than –0.6 (indicated by dashed gray line). All tests were performed with biological replicates as lactate (*n* = 5) and MACS (*n* = 5). Analysis was performed on hiPSC-CM cultures at day 24, with day 0 as the start of hiPSC differentiation. Lactate purification occurred from hiPSC-CM day 17 to day 24. MACS purification occurred at day 24 the day of proteomics analysis.

We identified over 4,000 unique proteins in each hiPSC-CM sample (Figure 2A; lactate = 4,478 ± 48 proteins [*n* = 5] versus MACS = 4,494 ± 51 proteins [*n* = 5]) (Supplemental Data R Notebook). Principal component analysis (PCA) of log_2_ protein abundances demonstrated clear separation between lactate and MACS purification by the main component (41.92%; Figure 2B). Differential protein analysis (adjusted *P* value [*P*_adj_] ≤ 0.05 and log_2_ fold change ≥ 0.6 for proteins with significant changes) revealed 707 differentially expressed proteins out of the 5,769 proteins identified per sample (413 proteins significantly increased in lactate hiPSC-CMs and 294 proteins significantly decreased in lactate hiPSC-CMs, compared with MACS hiPSC-CMs; Figure 2, C and D). Importantly, we found a similar reduction in cardiac sarcoplasmic reticulum Ca^2+^-ATPase2a (SERCA2a) expression (lactate versus MACS, log_2_ fold change: –0.601, *P*_adj_ = 0.00621) shown by Davis et al. (3) in the lactate hiPSC-CMs. Visualization of the top 50 differentially expressed proteins by *P* value revealed differences in other proteins involved in contraction, metabolism, and cellular remodeling (Figure 2E).

We performed pathway analyses using UniProt (https://www.uniprot.org/), Reactome (https://reactome.org/), and PANTHER (https://www.pantherdb.org/) databases to determine cellular pathway trends within differentially expressed proteins (44–47). Relevant keywords such as “muscle”, “cardiomyopathy”, “reactive oxygen species”, “glycolysis”, “beta-oxidation”, “microtubule”, “hypoxia”, “senescence”, “TGFβ”, “adrenaline”, “trafficking”, “hypertrophy,” and “mitochondrial” were used to identify the most relevant pathways from UniProt and Reactome. The top 12 pathway hits identified several differentially expressed pathways related to cellular metabolism, including a decrease in glycolysis (overall pathway expression < –0.6 log_2_ fold change expression) and an increase in mitochondrial fatty acid β-oxidation (overall pathway expression > 0.6 log_2_ fold change expression) in lactate hiPSC-CMs (Figure 2F). Glycolysis (GO: P00024) was also indicated by the PANTHER search as significantly downregulated in lactate hiPSC-CMs (Supplemental Figure 1). Additional changes in mitochondrial-related activity for lactate hiPSC-CMs were implicated by a significant increase in transcriptional activation of mitochondrial biogenesis. Posttranslational protein phosphorylation was also shown to be significantly upregulated (Figure 2F). While additional pathways indicated by Davis et al. (3) did not show overall differential pathway expression, such as degradation of the extracellular matrix and detoxification of ROS, individual proteins within each pathway were differentially expressed. Thus, our proteomics data reveal that lactate selection has profound effects on monolayer hiPSC-CMs directly after purification, producing a distinct molecular signature consistent with that observed by Davis et al. (3).

### hiPSC-ECT structure is consistent between lactate and MACS purification methods.

**Figure 3:**
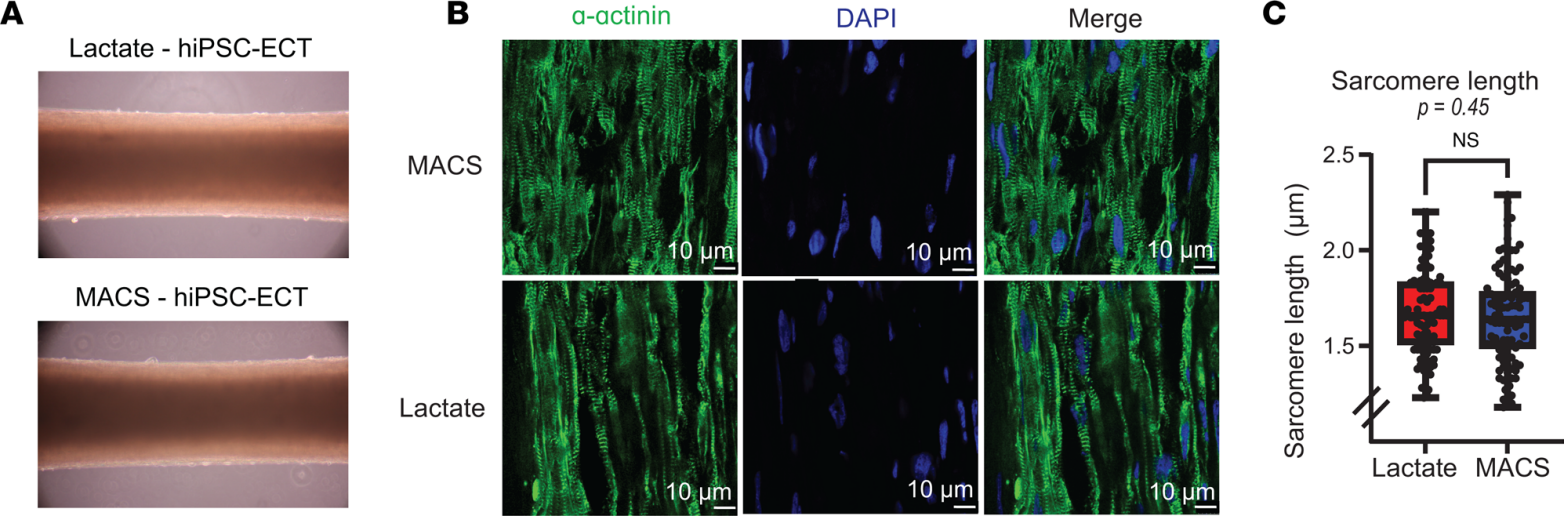
Sarcomere lengths of hiPSC-ECTs. (**A**) Three-dimensional representations of hiPSC-ECTs from lactate and MACS purification methods. Three-dimensional hiPSC-ECT images taken at day 58 prior to assessment with 50× Olympus microscope. (**B**) α-Actinin and DAPI immunofluorescence labeling on representative lactate and MACS hiPSC-ECTs. (**C**) Sarcomere length comparison (100 total measurements per condition, *P* = 0.45) using manual annotation of *Z* disc length via α-actinin staining. All tests were performed with biological replicates as lactate (*n* = 3) and MACS (*n* = 3), unless otherwise stated. All statistical analyses are 2-tailed Student’s *t* tests with α = 0.05. Plots show whiskers from 0 to 25th percentile, box from 25th to 75th percentile (mean indicated with line), and whiskers from 75th to 100th percentile. IHC was performed with hiPSC-ECTs at day 28, with day 0 being the generation of hiPSC-ECTs.

After confirming a differential phenotype in our 2D hiPSC-CMs, we sought to fully assess whether lactate purification affected CM structure or morphology after subsequent culture in 3D hiPSC-ECTs. After purification of monolayer cultures, we combined hiPSC-CMs in a 10:1 ratio (2 × 10^6^ hiPSC-CMs and 2 × 10^5^ fibroblasts) with low-passage isogenic hiPSC-CFs and seeded the cell mixture in a fibrinogen-thrombin matrix to form hiPSC-ECTs (48). No global morphological differences were observed between lactate and MACS hiPSC-ECTs (Figure 3A). We then cultured the hiPSC-ECTs for 4 weeks to allow for construct remodeling and maturation before subsequent analyses (ECT age before analysis from day 0 of ECT generation; lactate = 28.59 ± 0.5 days [*n* = 22] versus MACS = 27.36 ± 0.9 days [*n* = 14]; *P* = 0.21). We first assessed the myofibril structure of our hiPSC-ECTs using histological methods. After 4 weeks in culture, a cohort of hiPSC-ECTs was fixed in OCT, sectioned, and stained with α-actinin, a *Z* disc protein flanking the ends of the cardiac sarcomere, to assess sarcomere length (Figure 3B and Supplemental Figure 2) (49, 50). Three hiPSC-ECTs per condition were sectioned onto multiple slides to assess sarcomere structure and cellular alignment. Indeed, there was no statistical difference in sarcomere length between MACS and lactate hiPSC-ECTs (Figure 3C; lactate = 1.67 ± 0.02 μm [*n* = 99] versus MACS = 1.65 ± 0.02 μm [*n* = 100]; 2-tailed unpaired *t* test, *P* = 0.45). This analysis indicates that 2D hiPSC-CM purification methods do not affect myofibril organization when the cells are subsequently cultured over time in 3D hiPSC-ECTs.

### Lactate purification has no effect on hiPSC-ECT twitch force parameters compared with MACS hiPSC-ECTs.

**Figure 4:**
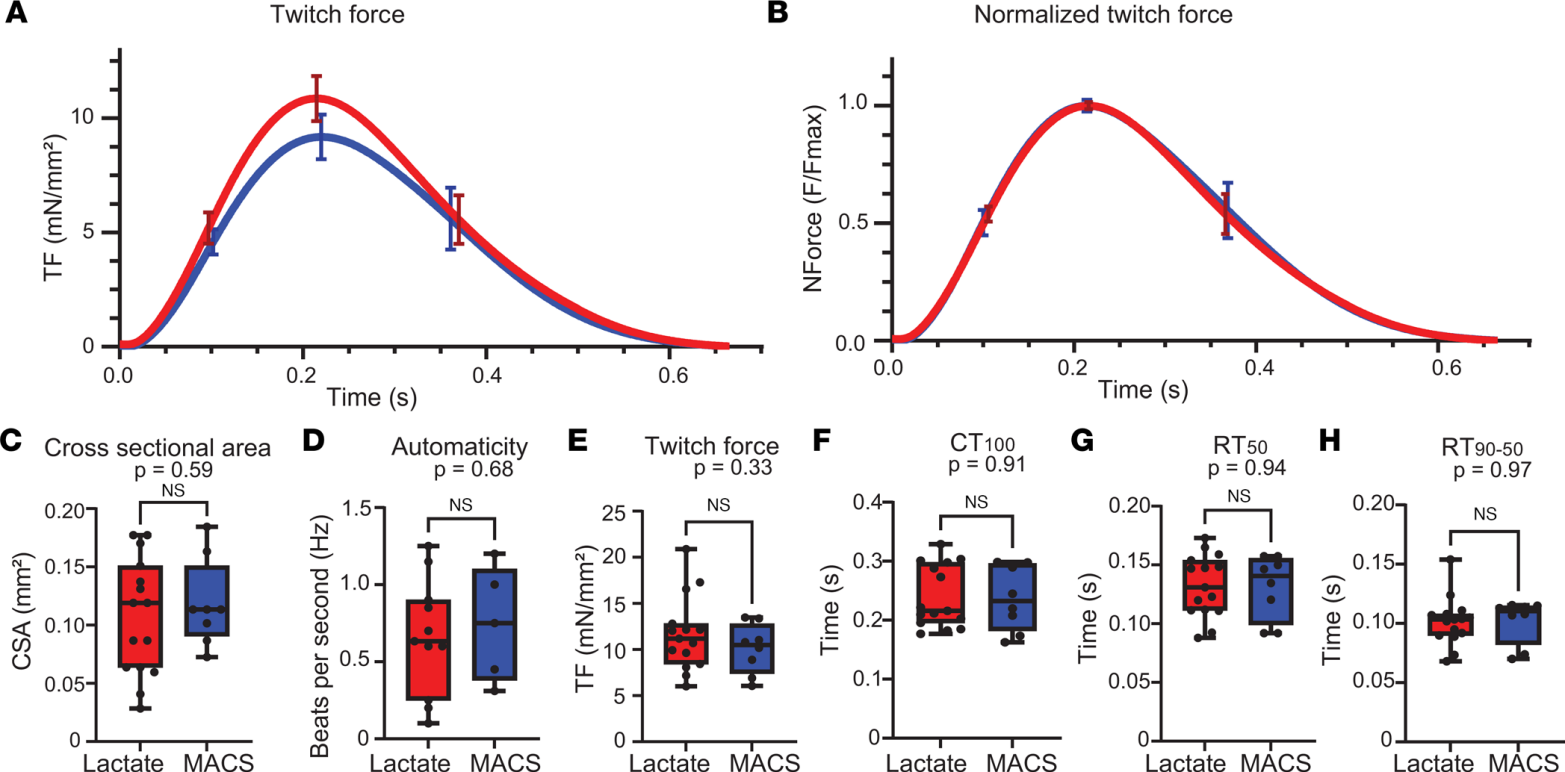
Twitch force assessment of hiPSC-ECTs. (**A** and **B**) Raw twitch force (TF) (**A**) and normalized averaged TF (NForce) (**B**) curves for lactate (red) and MACS (blue) hiPSC-ECTs. SEM at 25%, 50%, and 75% are shown for each curve. (**C**–**H**) TF parameters for lactate (red) and MACS (blue) hiPSC-ECTs as follows: cross-sectional area (CSA, *P* = 0.59) (**C**); automaticity (lactate, *n* = 11 versus MACS, *n* = 5;*P* = 0.68) (**D**); TF amplitude (*P* = 0.33) (**E**); time from pacing stimulus to TF peak (CT_100_, *P* = 0.91) (**F**); time from TF peak to 50% twitch force decay (RT_50_, *P* = 0.94) (**G**); and time from 50% to 90% TF decay (RT_50–90_; *P* = 0.97) (**H**). All tests were performed with biological replicates as lactate (*n* = 15) and MACS (*n* = 9), unless otherwise stated. All statistical analyses are 2-tailed Student’s *t* test with α = 0.05. Plots show whiskers from 0 to 25th percentile, box from 25th to 75th percentile (mean indicated with line), and whiskers from 75th to 100th percentile. Functional assays were performed with hiPSC-ECTs from days 26 to 31, with day 0 being the generation of hiPSC-ECTs.

After 4 weeks in 3D culture, we performed extensive functional characterization of the lactate and MACS hiPSC-ECTs. hiPSC-ECT constructs were electrically paced at 1.5 Hz in a 37°C physiologic chamber for the duration of the functional analyses. To determine baseline contractile function, we measured twitch force amplitude (TF), time from pacing stimulus to twitch force peak (CT_100_), time from twitch force peak to 50% twitch force decay (RT_50_), and time from 50% to 90% twitch force decay (RT_50–90_) (Figure 4 and Supplemental Figure 3A). Cross-sectional area (CSA) was not significantly different (Figure 4C; lactate = 0.11 ± 0.01 mm^2^ [*n* = 15] versus MACS = 0.12 ± 0.01 mm^2^ [*n* = 9]; 2-tailed unpaired *t* test, *P* = 0.59), indicating successful construct remodeling in both MACS and lactate hiPSC-ECTs.

Additionally, automaticity was not significantly different between enrichment conditions (Figure 4D; lactate = 0.66 ± 0.11 Hz [*n* = 11] versus MACS = 0.74 ± 0.17 Hz [*n* = 5]; 2-tailed unpaired *t* test, *P* = 0.68), and no arrythmias were observed while capturing automaticity, even after challenge with 1 μM isoproterenol (Supplemental Figure 4). TF was similar for lactate and MACS hiPSC-ECTs (Figure 4E; lactate = 11.7 ± 1.04 mN/mm^2^ [*n* = 15] versus MACS = 10.1 ± 0.96 mN/mm^2^ [*n* = 8]; 2-tailed unpaired *t* test, *P* = 0.33). Other twitch force parameters, such as CT_100_ (Figure 4F; lactate = 0.240 ± 0.014 seconds [s] [*n* = 15] versus MACS = 0.237 ± 0.019 s [*n* = 8]; 2-tailed unpaired *t* test, *P* = 0.91), RT_50_ (Figure 4G; lactate = 0.132 ± 0.007 s [*n* = 15] versus MACS = 0.131 ± 0.010 s [*n* = 8]; 2-tailed unpaired *t* test, *P* = 0.94), and RT_50–90_ (Figure 4H; lactate = 0.102 ± 0.005 s [*n* = 15] versus MACS = 0.102 ± 0.007 s [*n* = 8]; 2-tailed unpaired *t* test, *P* = 0.97), displayed similar ranges across hiPSC-ECTs and were not significantly different. Collectively, the contractile measurements indicate lactate hiPSC-ECTs display similar baseline functional properties compared with MACS hiPSC-ECTs.

### Intracellular calcium handling is similar between purification protocols.

**Figure 5:**
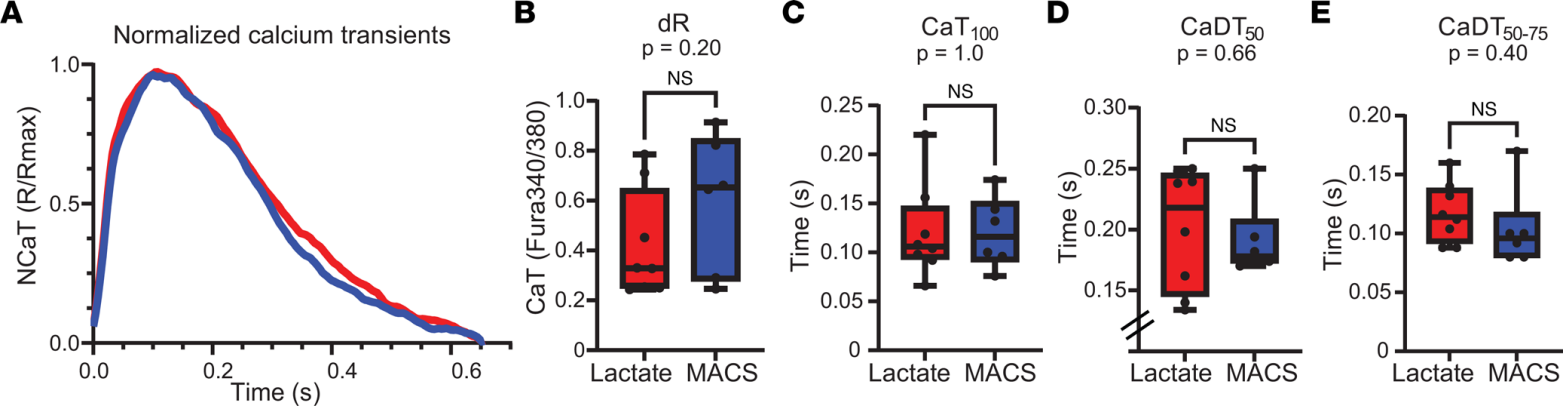
Calcium transients assessment of hiPSC-ECTs. (**A**) Normalized averaged calcium transients (NCa^2+^TR) curves for lactate (red) and MACS (blue) hiPSC-ECTs. (**B**–**E**) Ca^2+^TR parameters for lactate (red) and MACS (blue) hiPSC-ECTs as follows: Ca^2+^TR peak (dR, *P* = 0.20) (**B**); time to Ca^2+^TR peak (CaT_100_, *P* = 1.0) (**C**); time from Ca^2+^TR peak to 50% Ca^2+^TR decay (CaDT_50_, *P* = 0.66) (**D**); and time from 50% Ca^2+^TR decay to 75% Ca^2+^TR decay (CaDT_50–75_, *P* = 0.40) (**E**). All tests were performed with biological replicates as lactate (*n* = 8) and MACS (*n* = 6). All statistical analyses are 2-tailed Student’s *t* tests with α = 0.05. Plots show whiskers from 0 to 25th percentile, box from 25th to 75th percentile (mean indicated with line), and whiskers from 75th to 100th percentile. Functional assays were performed with hiPSC-ECTs from days 26 to 31, with day 0 being the generation of hiPSC-ECTs.

We then sought to understand how calcium handling may be affected in our lactate hiPSC-ECTs. Ca^2+^TR were assessed at 1.5 Hz using the Ca^2+^ indicator Fura2-AM (Figure 5 and Supplemental Figure 3B). All Ca^2+^ parameters were consistently similar between lactate and MACS hiPSC-ECTs, including Ca^2+^TR peak (Figure 5B; dR; lactate = 0.42 ± 0.08 Fura340/380 [*n* = 8] versus MACS = 0.60 ± 0.11 Fura340/380 [*n* = 6]; 2-tailed unpaired *t* test, *P* = 0.20), time to Ca^2+^TR peak (Figure 5C; CaT_100_; lactate = 0.120 ± 0.017 s [*n* = 8] versus MACS = 0.120 ± 0.015 s [*n* = 6]; 2-tailed unpaired *t* test, *P* = 1.0), time from Ca^2+^TR peak to 50% Ca^2+^TR decay (Figure 5D; CaDT_50_; lactate = 0.201 ± 0.018 s [*n* = 8] versus MACS = 0.191 ± 0.012 s [*n* = 6]; 2-tailed unpaired *t* test, *P* = 0.66), and time from 50% Ca^2+^TR decay to 75% Ca^2+^TR decay (Figure 5E; CaDT_50–75_; lactate = 0.118 ± 0.009 s [*n* = 8] versus MACS = 0.104 ± 0.014 s [*n* = 6]; 2-tailed unpaired *t* test, *P* = 0.40). These findings indicate that Ca^2+^ handling is similar between lactate and MACS hiPSC-ECTs.

### Global proteome of lactate hiPSC-ECTs is similar to MACS hiPSC-ECTs.

After functional characterization, we sought to assess the global expression profile arising from the same hiPSC-ECTs. Immediately following isoproterenol treatment, hiPSC-ECTs were flash frozen and stored at –80˚C until proteomic analysis. Azo (0.25% w/v) was used to solubilize the hiPSC-ECT global proteome similar to established protocols (43, 44). Aliquots from the protein homogenate were saved for SDS-page analysis of extraction reproducibility (Supplemental Figure 5), and injection similarity was evaluated using total ion chromatograms (Supplemental Figure 6).

**Figure 6:**
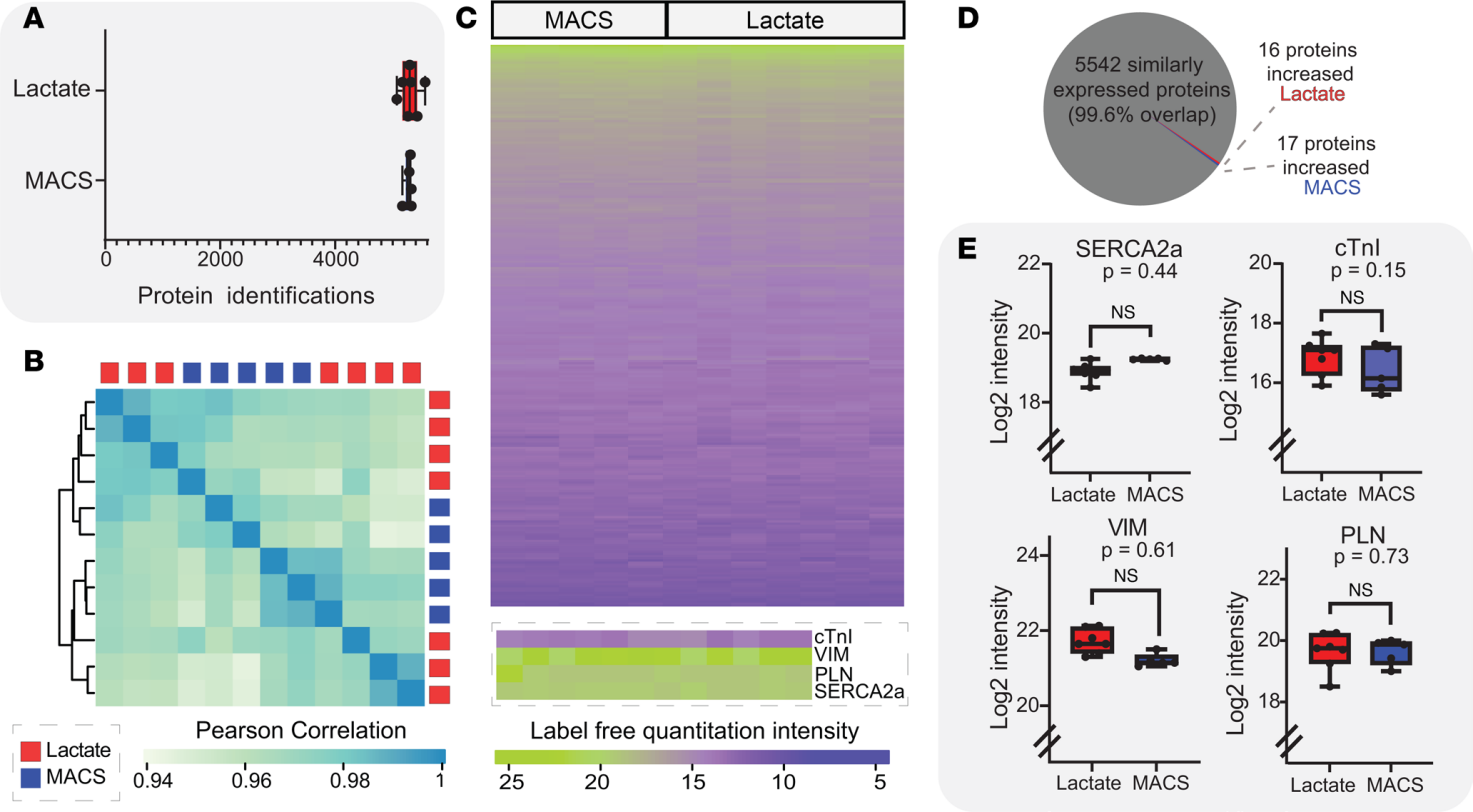
Global proteomics analysis of hiPSC-ECTs. (**A**) Unique protein identifications per hiPSC-ECT group. (**B**) Pearson correlation of hiPSC-ECT replicates with unbiased dendrogram clustering. (**C**) Heatmap of overall protein expression with expression of cardiac troponin I (cTnI), phospholamban (PLN), cardiac sarcoplasmic reticulum Ca^2+-^ATPase2a (SERCA2a), and vimentin (VIM) shown for each replicate. (**D**) Pie chart for visual representation of differentially expressed proteins (*P*_adj_ ≤ 0.05 and |log_2_ fold change| ≥ 0.6 required to be considered significant). (**E**) Log_2_ fold intensity values plotted for cTnI, PLN, SERCA2a, and VIM, as proteins of interest (cTnI, *P* = 0.15; PLN, *P* = 0.73; SERCA2a, *P* = 0.44; VIM, *P* = 0.61). All tests were performed with biological replicates as lactate (*n* = 7) and MACS (*n* = 5). All statistical analyses are 2-tailed Student’s *t* tests with α = 0.055. Plots show whiskers from 0 to 25th percentile, box from 25th to 75th percentile (mean indicated with line), and whiskers from 75th to 100th percentile. Proteomics assays were performed with hiPSC-ECTs from days 26 to 29, with day 0 being the generation of hiPSC-ECTs.

We identified over 5,000 unique proteins in each hiPSC-ECT sample (Figure 6A; lactate = 5,299 ± 60 proteins [*n* = 7] versus MACS = 5,262 ± 28 proteins [*n* = 5]) (Supplemental Data R Notebook). Pearson correlations of overall protein expression revealed the greatest deviation from linearity between any pair of hiPSC-ECT as *R* = 0.94. Unbiased hierarchical clustering of hiPSC-ECT over all protein expression indicated, via dendrogram, randomly grouped replicates between lactate and MACS hiPSC-ECTs (Figure 6B). Visualization of overall protein group expression between purification techniques by heatmap did not reveal any differentially expressed proteins (Figure 6C). Next, we performed differential expression analysis to identify proteins and/or pathways that were changing between hiPSC-ECT groups (*P*_adj_ = 0.05 or less and a log_2_ fold change ≥ 0.6 for proteins with significant changes). Our differential expression protein analysis revealed 5,542 proteins commonly expressed in all replicates with only 33 proteins being differentially expressed (99.6% similarity; Figure 6D and Supplemental Figure 7).

We performed pathway analyses using KEGG, UniProt/Reactome, and PANTHER databases to identify cellular and disease pathway trends within differentially expressed proteins (45–47). None of the databases indicated significantly changing pathways for the differentially expressed proteins. Therefore, we manually searched for overall pathway protein expression differences in our dataset using relevant keywords such as “muscle”, “cardiomyopathy”, “reactive oxygen species”, “glycolysis”, “beta-oxidation”, “microtubule”, “hypoxia”, “senescence”, “TGFβ”, “adrenaline”, “trafficking”, “hypertrophy,” and “mitochondrial”. These pathways were plotted with all identified proteins (Supplemental Figure 8). Overall, there was no significant changes in protein expression for the pathways identified. Key proteins identified by Davis et al. (3) as ischemic markers, including cardiac troponin I (cTnI), phospholamban (PLN), SERCA2a, and vimentin (VIM), were not differentially expressed (Figure 6E; cTnI; lactate = 16.86 ± 0.23 log_2_ fold value [*n* = 7] versus MACS = 16.41 ± 0.34 log_2_ fold value [*n* = 5]; 2-tailed unpaired *t* test, *P* = 0.15; PLN; lactate = 19.64 ± 0.23 log_2_ fold value [*n* = 7] versus MACS = 19.64 ± 0.19 log_2_ fold value [*n* = 5]; 2-tailed unpaired *t* test, *P* = 0.73; SERCA2a; lactate = 18.91 ± 0.10 log_2_ fold value [*n* = 7] versus MACS = 19.26 ± 0.01 log_2_ fold value [*n* = 5]; 2-tailed unpaired *t* test, *P* = 0.44; VIM; lactate = 21.72 ± 0.12 log_2_ fold value [*n* = 7] versus MACS = 21.21 ± 0.08 log_2_ fold value [*n* = 5]; 2-tailed unpaired *t* test, *P* = 0.61). Therefore, our proteomics data indicate that global proteome expression is unaffected by lactate purification in 3D cultures.

### Myofilament proteoform expression of lactate hiPSC-ECTs is comparable with MACS hiPSC-ECTs.

We then sought to evaluate the possible effect of lactate purification on myofibril organization through sarcomere protein expression and organization. We aimed to target possible protein isoform and phosphorylation changes within the proteins of the contractile apparatus using intact protein analysis. After baseline twitch force characterization only, a separate group of hiPSC-ECTs was flash frozen and stored at −80°C until targeted top-down proteomics analysis was performed as previously described (51). For myofilament analysis, constructs were homogenized in a lysis buffer containing HEPES (pH 7) to deplete cytosolic proteins. The sarcomere-enriched pellet was exposed to a trifluoroacetic acid (TFA) buffer (pH 2) to create a sarcomere-rich extract. Equal amounts of proteins from the TFA extract were analyzed by top-down liquid chromatography–tandem MS (LC-MS/MS) (Supplemental Figure 9; see complete unedited blots in the supplemental material).

**Figure 7:**
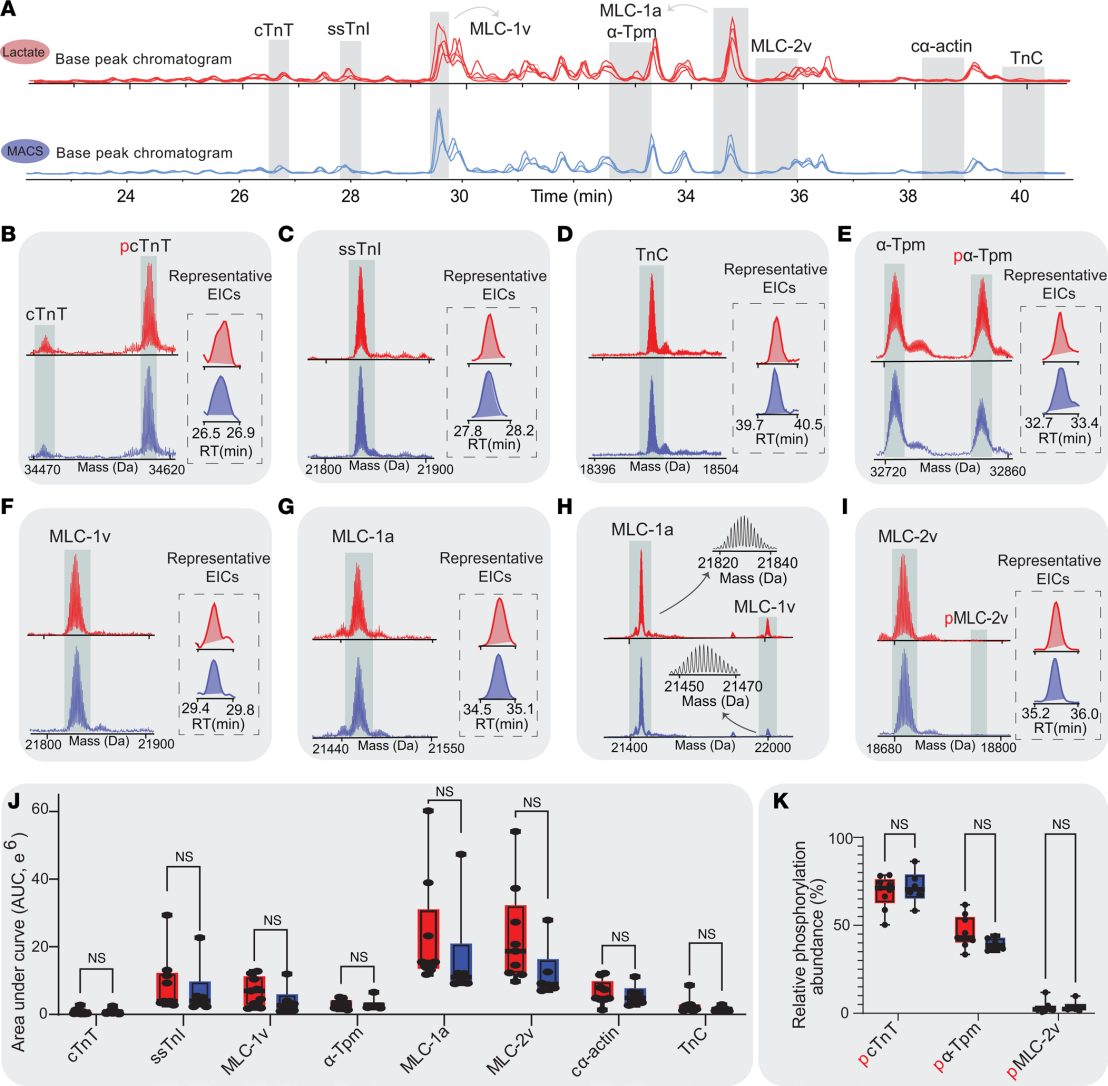
Top-down proteomics of the hiPSC-ECT cardiac sarcomere. (**A**) Selected base peak chromatograms for lactate and MACS replicates with identified proteins based on retention time (RT). (**B**–**I**) Spectra and isotopic resolution given for cardiac troponin T (cTnT) (**B**), slow skeletal troponin I (ssTnI) (**C**), troponin C (TnC) (**D**), α tropomyosin (α-Tpm) (**E**), myosin light chain 1v (MLC-1v) (**F**), myosin light chain 1a (MLC-1a) (**G**), MLC-1 isoforms (**H**), and myosin light chain 2v (MLC-2v) (**I**) with RT shown with extraction ion chromatograms (EICs). (**J**) Differential quantitation of myofilament proteins (cTnT, *P* = 0.94; ssTnI, *P* = 0.63; MLC-1v, *P* = 0.22; α-Tpm, *P* = 0.97; MLC-1a, *P* = 0.46; MLC-2v, *P* = 0.13; cα-actin, *P* = 0.62; TnC, *P* = 0.32). (**K**) Relative phosphorylation of myofilament proteins (pcTnT, *P* = 0.60; pα-Tpm, *P* = 0.10; pMLC-2v, *P* = 0.86). All tests were performed with biological replicates as lactate (*n* = 9) and MACS (*n* = 6), unless otherwise stated. All statistical analyses are 2-tailed Student’s *t* tests with α = 0.05. Plots show whiskers from 0 to 25th percentile, box from 25th to 75th percentile (mean indicated with line), and whiskers from 75th to 100th percentile. Proteomic assays were performed with hiPSC-ECTs from days 20 to 28, with day 0 being the generation of hiPSC-ECTs.

Our highly reproducible, top-down targeted proteomics allowed us to obtain a bird’s eye view of all the detectable PTMs and isoforms of sarcomere proteins in a single injection (Figure 7A and Supplemental Figures 10 and 11) (13, 51–53). Due to the ability of intact protein analysis to confidently differentiate between isoforms, we quantified several sarcomere isoforms to validate our global proteomics results using the AUC given by extracted ion chromatograms (EICs; Supplemental Table 1). Cardiac troponin T (cTnT) modulates the interaction between the thick and thin filaments in the presence of calcium (54). We did not identify significant changes in either the phosphorylation state or overall expression given as AUC (Figure 7, B, J, and K; lactate = 0.79 ± 0.03 pTotal [*n* = 9] versus MACS = 0.82 ± 0.04 pTotal [*n* = 6]; 2-tailed unpaired *t* test, *P* = 0.60; lactate = 0.80 × 10^5^ ± 0.3 × 10^5^ AUC [*n* = 9] versus MACS = 0.84 × 10^5^ ± 0.3 × 10^5^ AUC [*n* = 6]; 2-tailed unpaired *t* test, *P* = 0.94) (55). Slow skeletal troponin I (ssTnI) is a critical isoform in cardiac development and is often used in ratio to cTnI to evaluate model maturation (13, 56). While cTnI has been previously reported using our top-down method for hiPSC-ECTs, its expression was not detected with this cell line (51). Nevertheless, the relative expression of ssTnI did not change between hiPSC-ECT groups (Figure 7C; lactate = 9.0 × 10^6^ ± 2.7 × 10^6^ AUC [*n* = 9] versus MACS = 6.8 × 10^6^ ± 2.9 × 10^6^ AUC [*n* = 6]; 2-tailed unpaired *t* test, *P* = 0.63). Troponin C (TnC), was also not significantly different in expression (Figure 7D; lactate = 2.6 × 10^6^ ± 0.7 × 10^6^ AUC [*n* = 9] versus MACS = 1.6 × 10^6^ ± 0.3 × 10^6^ AUC [*n* = 6]; 2-tailed unpaired *t* test, *P* = 0.32). Unphosphorylated and phosphorylated states of contractile thin filament protein α-tropomyosin (α-Tpm) were identified in both lactate and MACS hiPSC-ECTs. α-Tpm was the only Tpm isoform detected, and the total expression and phosphorylation of α-Tpm was not significantly different by pTotal calculations (Figure 7E; lactate = 3.0 × 10^6^ ± 0.4 × 10^6^ AUC [*n* = 9] versus MACS = 3.0 × 10^6^ ± 0.7 × 10^6^ AUC [*n* = 6]; 2-tailed unpaired *t* test, *P* = 0.97; lactate = 0.53 ± 0.03 pTotal [*n* = 9] versus MACS = 0.45 ± 0.01 pTotal [*n* = 6]; 2-tailed unpaired *t* test, *P* = 0.10). Differential expression of myosin light chain (MLC) isoforms can often indicate differences in maturation state (13). We did not identify significant changes in any MLC isoforms (Figure 7, F–H), including MLC-1v (lactate = 6.8 × 10^6^ ± 1.3 × 10^6^ AUC [*n* = 9] versus MACS = 3.9 × 10^6^ ± 1.5 × 10^6^ AUC [*n* = 6]; 2-tailed unpaired *t* test, *P* = 0.22), MLC-1a (lactate = 2.3 × 10^7^ ± 5.1 × 10^6^ AUC [*n* = 9] versus MACS = 1.7 × 10^7^ ± 5.6 × 10^6^ AUC [*n* = 6]; 2-tailed unpaired *t* test, *P* = 0.46), or their isoform ratio (lactate = 0.31 ± 0.04 AUC MLC-1v/1-a [*n* = 9] versus MACS = 0.22 ± 0.03 AUC MLC-1v/1-a [*n* = 6]; 2-tailed unpaired *t* test, *P* = 0.19). MLC-2v is a crucial component of the myosin motor and is directly involved in sarcomere contraction (57). Its emerging expression is indicative of a “mature” ventricular-like phenotype in hiPSC-CMs (13, 42). As anticipated, the expression of MLC-2v and phosphorylated isoform was comparable between lactate and MACS hiPSC-ECTs (Figure 7I; lactate = 2.30 × 10^7^ ± 4.6 × 10^6^ AUC [*n* = 9] versus MACS = 1.22 × 10^7^ ± 2.9 × 10^6^ AUC [*n* = 6]; 2-tailed unpaired *t* test, *P* = 0.13; lactate = 0.35 ± 0.01 pTotal [*n* = 9] versus MACS = 0.39 ± 0.01 pTotal [*n* = 6]; 2-tailed unpaired *t* test, *P* = 0.10). Additional contractile proteins, such as cardiac α-actin (cα-actin), were not significantly different in expression (Figure 7J and Supplemental Figure 12; lactate = 6.6 × 10^6^ ± 1.1 × 10^6^ AUC [*n* = 9] versus MACS = 5.6 × 10^6^ ± 1.2 × 10^6^ AUC [*n* = 6]; 2-tailed unpaired *t* test, *P* = 0.62). The absence of significant functional differences between hiPSC-ECTs formed from either lactate- or MACS-purified hiPSC-CMs is supported at the level of the proteome.

## Discussion

Lactate purification of hiPSC-CMs for subsequent use in 2D and 3D modeling has been widely adopted by the cardiovascular research community (18). Davis et al. (3) recently provided data suggesting that lactate purification of 2D hiPSC-CMs generates a phenotypically “ischemic” cell population, implying an adverse effect of metabolic selection on cells and, therefore, negative implications for their use in certain downstream applications. This is a critical issue since the purified hiPSC-CMs are being utilized by many groups to explore often subtle biological questions (19–39). Given the proven benefits of growing hiPSC-CMs in an integrated 3D tissue environment, we explored whether the 3D ECT would mitigate the functional issues observed in 2D-cultured lactate-purified hiPSC-CMs compared with the MACS-purification approach. Our detailed structural, functional, and proteomic comparison of 3D hiPSC-ECTs generated using either lactate- or MACS-purified hiPSC-CMs demonstrated a high degree of similarity between groups. Both hiPSC-ECT cohorts were structurally similar at the level of the visible hiPSC-ECT down to the myofilament level. Functionally, lactate hiPSC-ECTs demonstrated similar contractile properties and calcium handling compared with our MACS hiPSC-ECTs. Additionally, no arrythmias were observed throughout functional evaluation, even after β-adrenergic stimulation. Changes across the proteome — including those indicated in lactate-purified hiPSC-CM monolayers — and alterations in myofilament proteoforms were not detected.

Our global proteomics analysis of monolayer hiPSC-CMs directly after purification indicate similar differences in proteins involved in mitochondrial-related processes, sarcomere contraction, and calcium handling as indicated by Davis et al. (3). Rupert et al. (58) implicate that lactate selection of hiPSC-CMs caused an increase in oxidative phosphorylation when compared with nonselected hiPSC-CMs, which could represent subtle differences in culture media and experimental timing. Irrespective of the specific differential phenotype caused by lactate purification, the dramatic differences we observed in the global proteome of monolayer lactate hiPSC-CMs, including metabolic associated proteins, are resolved in our hiPSC-ECTs.

Remodeling of the 3D hiPSC-ECT construct is a critical component to successful model function and can be determined visually using a microscope (12, 59). We did not observe any differences in structure between the hiPSC-ECT groups at the macrolevel. A decrease in sarcomere length has been observed previously in animal models with myocardial infarction, specifically within the ischemic regions of the heart (60). Indeed, we observed no significant changes in sarcomere length, and this is in direct contrast to the 2D hiPSC-CM presentation observed by Davis et al. (3).

Functionally, while previous studies indicate lactate-purified hiPSC-CMs in 2D monolayer cultures demonstrate arrythmias and poor calcium handling 5 days after purification (similar to what has been found in CMs isolated from ischemic human hearts) (3, 61–63), our lactate hiPSC-ECTs demonstrated little variability from our MACS hiPSC-ECTs after 4 weeks in culture. Unlike stress or ischemic heart tissue, the lactate hiPSC-ECTs did not display arrythmias in β-adrenergic responsiveness (63). Most importantly, we demonstrate that lactate-generated hiPSC-ECTs retain full contractile function. This ability to directly measure force and contractile kinetics in an integrated human tissue is a great advantage of 3D hiPSC-CM models. By demonstrating an unaltered phenotype between lactate- and MACS-purified hiPSC-ECTs, we ensure there is no bias in our 3D models when testing multiple aspects of function, regardless of our chosen 2D hiPSC-CM purification method.

An important finding of our study is recovery of the lactate hiPSC-CM global proteome in 3D hiPSC-ECTs. Changes indicative of an ischemic phenotype — which often include decreased SERCA2a expression, reduction of mitochondrial- and metabolism-related protein pathways, and CM cytoskeletal changes — were not observed in the lactate-purified hiPSC-ECTs (3, 61, 64, 65), even though many of these molecular signatures were implicated in the lactate hiPSC-CMs directly following purification. Differentially expressed individual proteins in our hiPSC-ECTs accounted for 0.4% of the proteome identified and did not suggest alterations in any major protein pathways known to be related to ischemia. Indeed, one of the advantages in using 3D hiPSC-ECTs includes molecular signatures of a more mature physiological state (13). By demonstrating that there is no molecular bias in our lactate hiPSC-ECTs, we ensure this advantage to the 3D model remains. Previous studies in both 2D and 3D hiPSC-CM models have indicated both global protein expression and sarcomere phosphorylation as key markers for maturation (12, 13, 51). These data imply that lactate purification does not alter the maturation state of hiPSC-ECTs.

Additionally, myofilament proteoforms, including PTMs and isoforms, are unaltered between the hiPSC-ECT groups. Both sarcomere phosphorylation and isoform expression for key proteins were found to be similar between lactate and MACS hiPSC-ECTs. It has been shown that bottom-up proteomics analyses, which digest proteins into peptides prior to MS analysis, can obscure posttranslational and protein isoform information due to the peptide-to-protein inference problem (41, 53). We have employed top-down proteomics, which is a powerful technology for detection and quantification of PTMs and isoforms simultaneously in 1 spectrum (13, 51, 53). Notably, the atrial form of MLC-2a (*MYL7*) was significantly increased in the MACS hiPSC-ECTs in our global bottom-up experiments, whereas in our top-down data set, MLC-2a was not detected at all. Given the sensitivity and accuracy of our top-down platform and the important role MLC has in development and maturation, this result emphasizes the importance of performing intact protein measurements to differentiate protein isoforms (13, 56, 66, 67). Here, we have shown that there are no alterations in the sarcomeric protein PTMs and isoforms between lactate and MACS hiPSC-ECTs; this implies a similar baseline molecular state for the sarcomere.

Taken together, these data indicate that there is no difference in structure, function, or proteome between lactate and MACS hiPSC-ECTs. Whether the absence in our study of the ischemic phenotype observed by Davis et al. in hiPSC-CM is due to the 3D culture configuration, extended time in culture, or both, is beyond the scope of this study (3). Rupert et al. (58) similarly reported analogous functionality between lactate-purified (82% cTnT^+^ population) and unpurified (38% cTnT^+^ population) 3D hiPSC-CM constructs after 1 week in culture. Another similar study (68) demonstrated effects on calcium handling and metabolism between lactate-conditioned (63.0% cTnT^+^ population) and unpurified (71.6% cTnT^+^ population) 3D hiPSC-CM constructs after 2 weeks in culture. These results may be indicative of varying miscellaneous cell populations, experimental design, and culture conditions. Furthermore, it should be noted that the 2D hiPSC-CMs experiments performed by Davis et al. (3) were within 5 days after selection and, therefore, may have not allowed for sufficient recovery of the hiPSC-CMs after lactate purification. Indeed, while consistency in differentiation, purification method, and inclusion of appropriate controls are all critical within an experimental design, the method of purification between lactate and MACS does not have a notable effect when hiPSC-CMs are subsequently grown in ECTs.

## Methods

### hiPSC culture.

Control hiPSCs were provided by the Stanford Cardiovascular Institute (Stanford University Cardiovascular Institutes Biobank, Stanford, CA, USA; Hypertrophic Cardiomyopathy/*MYH7*/Control iPSC line) (4). All hiPSCs were maintained in StemFlex media (Thermo Fisher Scientific) according to the manufacturer’s protocol. Cryopreserved hiPSCs were thawed and added to StemFlex media supplemented with 5 μM Y-27632 (BD Biosciences). hiPSCs were then plated onto 8.7 μg/cm^2^ Matrigel-coated (GFR, BD Biosciences) 6-well dishes. hiPSCs were subsequently cultured at 37°C at 5% CO_2_ until they were 70%–90% confluent with daily media changes before passaging. For passaging, hiPSCs were dissociated using Versene (Thermo Fisher Scientific), resuspended in StemFlex media, and replated onto Matrigel-coated plates (Corning).

### Differentiation of hiPSCs into CMs.

hiPSCs from the Stanford Cardiovascular Institutes (SCVI) WT line were differentiated into CMs using a small molecule–directed protocol as previously described (42). In short, hiPSCs in culture were dissociated and seeded onto Matrigel-coated 6-well plates at 1.5 × 10^6^ to 2.0 × 10^6^ cells/well in StemFlex media. Cells were cultured for approximately 3–5 days in StemFlex media (1 day after 100% confluence, at which time differentiation was initiated [day 0]). On day 0, StemFlex media were replaced with RPMI supplemented with B27 without insulin (Thermo Fisher Scientific) with a 9 μM CHIR99021 addition (Tocris Bioscience). Exactly 24 hours later (day 1), media were changed to 3 mL/well RPMI + B27 without insulin and cells were cultured in this media for 48 hours (day 3). On day 3, the media were changed to 3 mL/well RPMI + B27 without insulin supplemented with 5 μM IWP-2 (Stemgent). Forty-eight hours later (day 5), the media were changed to 3 mL RPMI + B27 without insulin. The media were changed to RPMI + B27 complete supplement (with insulin) (Thermo Fisher Scientific) on day 7, and the differentiated cells were maintained in this media until day 15, with media changes every 48–72 hours. On day 15, cells from wells containing ≥ 80% contracting cells by visual inspection were dissociated with 10× TrypLE (Thermo Fisher Scientific) according to the manufacturer’s protocol. Cells were then cryopreserved using a 1:9 ratio of DMSO (MilliporeSigma) and FBS (Thermo Fisher Scientific).

### hiPSC-CM purification.

After thawing and suspending in EB20 media (2), cells were replated on Synthemax-coated (Corning) 6-well plates. Each hiPSC-CM batch (5 batches of 15 million cells) was matched for both lactate and MACS purifications. For the lactate-purified hiPSC-CMs, 48 hours after replating, hiPSC-CMs were purified using CDM3L media, made with RPMI 1640 no glucose (Invitrogen), 500 μg/mL recombinant human albumin, 213 μg/mL L-ascorbic acid 2-phosphate, and 4 mM L-lactic acid (Sigma-Aldrich) (16) for 7 days, with media changes every 48–72 hours. Following selection, hiPSC-CMs were maintained in RPMI with B27 supplement until day 30, at which point hiPSC-CMs were combined with hiPSC-CFs to generate ECTs. For the MACS-selected cells, thawed cells were maintained in RPMI with B27 supplement until day 30, which is the day of ECT generation. Cells were dissociated from the plate using 10× TrypLE (Thermo Fisher Scientific) and purified with the MACS system (Miltenyi Biotec) according to the manufacturer’s protocol. In summary, the cell suspension was quenched with EB20, passed through a 100 μM cell strainer (Thermo Fisher Scientific), counted, and centrifuged (200*g* at 4°C for 5 minutes). The remaining cell pellet was then resuspended (80 μL/5 × 10^6^ cells) in “Miltenyi” buffer containing 0.5% w/v BSA (MilliporeSigma), DPBS, and 2 mM EDTA (Thermo Fisher Scientific). Non-Cardiomyocyte Depletion Cocktail (Miltenyi Biotec) was added (20 μL/5 × 10^6^ cells) and incubated at 4°C for 5 minutes. The mixture was then washed with additional Miltenyi buffer (0.5% BSA w/v [MilliporeSigma] with 4% EDTA 0.5M solution [pH 8.0; Thermo Fisher Scientific] in DPBS, no Ca or Mg [Thermo Fisher Scientific]) and centrifuged (200*g* at 4°C for 5 minutes). The cell pellet was then resuspended (80 μL/5 × 10^6^ cells) in Miltenyi buffer, and anti-biotin MicroBeads (Miltenyi Biotec) were added in a 20 μL/5 × 10^6^ cell ratio and incubated at 4˚C for 10 minutes. Additional buffer was added to bring the final concentration to 500 μL/5 × 10^6^ cells. A total of 500 μL of the cell suspension was loaded into the LS Column/MACS Separation apparatus (Miltenyi Biotec). Flow-through of the pure hiPSC-CMs was centrifuged (200*g* at 4°C for 5 minutes), resuspended, and taken for either ECT generation or flow cytometry analysis. Images of hiPSC-CMs were taken using 40× Olympus microscope objective via DSLR camera mounted to a cell culture microscope. Images were not digitally altered in any way.

### Differentiation of hiPSCs into fibroblasts.

Isogenic hiPSC-CFs were generated as previously described (48). In brief, hiPSCs were dissociated with Versene solution (Thermo Fisher Scientific) and seeded on Matrigel-coated 6-well plates at the density of 1.5 × 10^6^ to 2.0 × 10^6^ cells/well in mTeSR1 media (WiCell) supplemented with 10 μM Y-27632. Cells were maintained in mTeSR1 media for approximately 5 days with daily changes until they reached 100% confluency (day 0). On day 0, the cells were treated with 2 mL RPMI + B27 without insulin and supplemented with 12 μM CHIR99021 for 24 hours. After 24 hours, the media were changed to 2 mL RPMI + B27 without insulin for 24 hours. After 24 hours, the media were changed to 2 mL of the CFBM media with 75 ng/mL bFGF (WiCell). Subsequently, cells were maintained with CFBM + 75 ng/mL bFGF every other day until day 20. On day 20, cells were either taken for flow cytometry analysis or cryopreserved using a 1:9 ratio of DMSO (MilliporeSigma) and FBS (Thermo Fisher Scientific). Once thawed, the hiPSC-CFs were maintained in FibroGRO-LS media (MilliporeSigma) in uncoated 6-well culture plates (Corning) and passaged every 5–6 days. Low passage number (<12 passages) were used for hiPSC-ECT generation.

### Flow cytometry.

hiPSC-CMs and hiPSC-CFs were analyzed as previously described (42, 48). Briefly, dissociated cells were vortexed to disrupt the aggregates; neutralization followed, as equal volume of EB20 media were added. Cells were counted to designate 1 million cells for labeling. Cells were fixed in 1% paraformaldehyde, washed with FACS buffer (DPBS, 0.5% BSA, 0.1% NaN_3_), centrifuged (200*g* at 4°C for 5 minutes), and resuspended in about 50 μL FACS. Primary antibodies, including monoclonal anti–α-actinin (IgG1, MilliporeSigma, A7732, 1:500 dilution) and monoclonal anti-cTnT (IgG1, Thermo Fisher Scientific, A-21121, 1:200 dilution), were incubated with the cells according to the manufacturer’s instructions. Afterward, cells were washed with FACS buffer plus 0.1% Triton X-100 and centrifuged (200*g* at 4°C for 5 minutes), and all but 50 μL supernatant was discarded. Secondary antibody appropriate to the primary IgG isotype (IgG, Thermo Fisher Scientific, MS-113-P1; IgG1, Thermo Fisher Scientific, MS295P) and was diluted at 1:1,000 in FACS buffer plus 0.1% Triton X-100. Samples were incubated for 30 minutes at room temperature, washed in FACS buffer, and resuspended in FACS buffer for analysis. Data were collected on a FACSCalibur (Beckton Dickinson) and Attune Nxt (Thermo Fisher Scientific) flow cytometers and were analyzed using FlowJo.

### hiPSC-ECT generation.

Day 30 lactate-purified hiPSC-CMs were dissociated with 10× TrypLE and counted using a hemocytometer. MACS-purified hiPSC-CMs were taken straight from purification and counted using hemocytometry. hiPSC-CMs were subsequently resuspended at 2 × 10^6^ CM/mL in fibrin ECT media (60.3% high-glucose DMEM; 20% F12 nutrient supplement; 1 mg/mL gentamicin; 8.75% FBS; 6.25% horse serum; 1% HEPES; 1× nonessential amino acid cocktail; 3 mM sodium pyruvate; 0.004% [wt/vol] NaHCO_3_; 1 μg/mL insulin; 400 μM tranexamic acid; and 17.5 μg/mL aprotinin) (13) and incubated for at least 1.5 hours on a rotating platform at 37°C. Low-passage isogenic hiPSC-CFs were dissociated using 1× TrypLE (Thermo Fisher Scientific) and counted using a hemocytometer. After rotational culture, hiPSC-CMs were mixed with hiPSC-CFs in a 10:1 ratio per hiPSC-ECT, as previously described (69). In total, 1.25 mg/mL fibrinogen and 0.5 unit of thrombin were added to the cell mixture, and the cell suspension was quickly loaded onto a 20 × 3 mm cylindrical mold of FlexCell Tissue Train silicone membrane culture plate. Following polymerization of the fibrin matrix, ECTs were maintained with fibrin ECT media at 37°C with 5% CO_2_ for 4 weeks, with media changes every 2–3 days. Images of hiPSC-ECTs were taken using 5× Olympus microscope objective via microscope attachment on Sony digital camera. Images were not digitally altered in any way.

### Twitch force and Ca^2+^TR measurements.

Isometric twitch force and Ca^2+^TR were measured in hiPSC-ECT using protocols similar to those previously described (13, 70). In brief, each hiPSC-ECT construct was attached using sutures to a model 801B small intact fiber test apparatus (Aurora Scientific) in Krebs-Henseleit buffer (119 mmol/L NaCl, 12 mmol/L glucose, 4.6 mmol/L KCl, 25 mmol/L NaHCO_3_, 1.2 mmol/L KH_2_PO_4_, 1.2 mmol/L MgCl_2_, 1.8 mmol/L CaCl_2_, gassed with 95% O_2_ to 5% CO_2_ [pH 7.4]) at 37°C. Krebs-Henseleit buffer flowed throughout the experiments at a rate of 1 mL/min. Force readouts were performed on a model 403A force transducer (Aurora Scientific). Stimulation was initiated on each hiPSC-ECT at 1 Hz (2.5 ms, 12.5 V). Constructs were stretched incrementally by 0.12 mm until plateau TF was achieved. Each construct was stretched to optimal length (until maximal twitch force was achieved based on Frank-Starling law; approximately 2.2 μm sarcomere length). Constructs were left to equilibrate for 20 minutes at 1 Hz. Following equilibration, twitch force production was measured with pacing at a frequency of 1.5 Hz both at baseline and following 5-minute preincubation with 1 μM isoproterenol. Automaticity was captured after pacing. Functional data generated from ECTs producing > 250 μN of raw twitch force were included in the analysis. After functional analyses, hiPSC-ECTs were washed with DPBS, flash frozen, and stored at –80˚C until proteomic analysis.

hiPSC-ECTs were then introduced to a Fura-2 loading solution consisting of Krebs-Henseleit buffer supplemented with 5 μM Fura2-AM (Invitrogen) and 1% (vol/vol) Chremophor EL (MilliporeSigma) with constant oxygenation (95% O_2_, 5% CO_2_) for 30 minutes at 37°C. Following Fura-2 loading, ECTs were left to equilibrate for 40 minutes with perfusion with Krebs-Henseleit buffer at a rate of 1 mL/min and paced at 1 Hz. Both twitch force and Ca^2+^TR data were recorded at pacing frequency 1.5 Hz. Fura-2 fluorescence was measured by alternately illuminating the preparation with 340 and 380 nm light (at a frequency of 250 Hz) while measuring the emission at 510 nm using IonOptix hardware and software (IonOptix Corporation). The emitted fluorescence and force data were stored as the 340 and 380 nm counts and as the ratio *R* = F340/F380. Data were analyzed using IonWizard 6.0 software (IonOptix). Under each condition, 40–60 successive contractions were collected and averaged. These data were exported to Microsoft Excel for parameter calculations. Statistical significances were determined by normal 2-tailed *t* test with α = 0.05 with 2-sided analysis. All data represented as data mean ± SEM.

### Cryopreservation, sectioning, and IHC.

hiPSC-ECTs were rinsed in DPBS (Thermo Fisher Scientific) in the well and incubated in 30 mM 2,3-butanedione monoxime in DPBS for 5 minutes. hiPSC-ECTs were then exposed to a filtered 30% sucrose (in DPBS) solution for 1 hour at room temperature, followed by a 1-hour incubation in a 1:1 mixture of optimal cutting temperature (OCT) compound (Tissue-Tek) and 30% sucrose solution in DPBS. The sucrose solution was aspirated from the well and hiPSC-ECTs were covered with OCT compound in the well before freezing the wells on a metal plate on dry ice. Cryopreserved hiPSC-ECTs were then stored at −80°C. The cryopreserved hiPSC-ECT disks were taken out of the plate before sectioning. Cryopreserved hiPSC-ECTs were then sectioned lengthwise at 6 μm thickness (Leica CM 1950UV), mounted onto charged slides (Superfrost +, Thermo Fisher Scientific), and fixed in 100% acetone for 15 minutes at 4°C. After drying, slides were placed in a vertical washer and rinsed with water for 10 minutes. Slides were then rehydrated in PBS and incubated in blocking buffer (0.15% Triton-X-100, 5% normal goat serum [NGS], 2 mg/mL BSA in PBS) for 1 hour at room temperature. Sections were incubated with primary antibodies overnight in a humidified chamber at 4°C with α-actinin (1:1,000, MilliporeSigma, A7811). Slides were incubated with secondary antibody (Alexa Fluor Plus 488; Invitrogen) at 5–8 μg/mL in blocking buffer for 1 hour at room temperature in a humidified chamber. Following labeling, sections were coverslipped using Prolong Gold Antifade Reagent (Invitrogen) with DAPI to label nuclei. Imaging was performed using Leica SP8 Confocal WLL STED Microscope using the 100× objective and Leica imaging software. Multiple sections from each hiPSC-ECT were imaged, and 100 sarcomeres were measured using manual annotation. The *Z* disc interval given by α-actinin staining was quantified via ruler function on the Leica Imaging software using the repeating fluorescence pattern of the α-actinin staining. Statistical significances were determined by normal 2-tailed *t* test with α = 0.05 with 2-sided analysis. All data represented as data mean ± SEM.

### Global bottom-up proteomics.

Global proteomics was performed similarly as previously described (71). Samples were randomized and blindly prepared to avoid bias. Cryopreserved hiPSC-ECTs or hiPSC-CMs were gently thawed on ice and homogenized in 40 μL of Azo (46) buffer (0.1% w/v Azo, 25 mM ammonium bicarbonate, 10 mM L-methionine, 1 mM dithiothreitol (DTT), and 1× HALT protease and phosphatase inhibitor) using a handheld Teflon homogenizer (Thomas Scientific). Samples were centrifuged (21,000*g* at 4°C for 15 minutes), and the supernatant was normalized to 1 mg/mL using 0.1% Azo buffer by the Bradford assay (Bio-Rad). Samples were reduced with 30 mM DTT for 1 hour and subsequently alkylated with 30 mM iodoacetamide for 1 hour. Trypsin Gold (Promega) was added in a 50:1 ratio to the samples and incubated for 24 hours. After 24 hours, trypsin was quenched with formic acid. Azo was then degraded at 305 nm for 5 minutes (Analytik Jena). Samples were centrifuged (21,000*g* at 4°C for 15 minutes), and the resulting supernatant was desalted using 100 μL Pierce C18 tips (Thermo Fisher Scientific) according to the manufacturer’s protocol. The peptides were dried in a vacuum centrifuge (21,000*g* at room temperature for 2 hours) and resuspended in 0.1% FA. The peptide concentrations were determined using Nanodrop One Microvolume UV-Vis Spectrophotometer (Thermo Fisher Scientific).

LC–trapped ion mobility spectrometry–tandem MS (LC-TIMS-MS/MS) was performed using a nanoElute nanoflow LC system (Bruker Daltonics) coupled to the timsTOF Pro (Bruker Daltonics). In total, 200 ng of each peptide sample was loaded on an Aurora Elite capillary C18 column (IonOpticks). Peptides were separated using a 120-minute gradient at a flow rate of 400 nL/min (mobile phase A [MPA]: 0.1% FA; MPB: 0.1% FA in acetonitrile). For the first 60 minutes, a gradient of 2%–17% MPB was applied, then 17%–25% MPB was applied for the next 30 minutes, 25%–37% MPB for 10 minutes, 37%–85% MPB for 10 minutes, and 85% MPB for an additional 10 minutes. The column utilized nanoESI source for sample passage to the mass spectrometer. MS spectra were captured with a Bruker timsTOF Pro quadrupole-TOF (Q-TOF) mass spectrometer (Bruker Daltonics) operating in diaPASEF mode, using 32 windows ranging from *m/z* 400 to 1,200 and 1/K_0_ 0.6–1.42. Data processing occurred similarly as previously described (72). LC-MS data were processed using DIA-Neural Network (DIA-NN) (73) using the default parameters unless noted in the following: 1% FDR, library-free search enabled; minimum fragment *m/z*, 200; maximum fragment *m/z*, 1,800; minimum precursor *m/z*, 400; maximum precursor *m/z*, 1,200; minimum precursor charge, 2; maximum precursor charge, 4; minimum peptide length, 7; maximum peptide length, 30; maximum missed cleavages, 2; MS1/MS2 mass accuracy, 10 ppm; quantification strategy, Robust LC (High Precision); NN classifier, souble-pass mode. All other analyses were performed in R. Protein-level quantification data were filtered using the “DAPAR” package (74) to include proteins identified in 2 of 3 runs in at least 1 sample group. Values were then median normalized, and missing values were imputed via ssla for partially observed values within a condition or set to the 2.5% quantile of observed intensities for observations that were missing entirely within a condition. The “DEP” R package was used to perform a Limma test between all specified contrasts, and the “IHW” R package was used to adjust all *P* values, using the number of quantified peptides per protein as a covariate. A *P*_adj_ threshold of 0.05 and a log_2_ fold change threshold of 0.6 were set to identify significant changes to protein abundance. Subsequent data were visualized and plotted using ggplot2 (74). All data represented as data mean ± SEM.

### Myofilament top-down proteomics.

Myofilament proteomics was performed similarly as previously described (51). All steps were kept at 4°C to minimize artifactual modifications (52). In summary, cryopreserved hiPSC-ECTs were gently thawed on ice and homogenized in 50 μL of HEPES extraction buffer (25 mM HEPES [pH 7.4], 60 mM NaF, 1 mM L-methionine, 1 mM DTT, 1 mM PMSF in isopropanol, 1 mM Na_3_VO_4_ containing protease and phosphatase inhibitors) using a handheld Teflon homogenizer. The cell homogenate was centrifuged (21,000*g* at 4°C for 30 minutes), and the supernatant containing cytosolic proteins was discarded. The resulting pellet was washed again in HEPES extraction buffer and centrifuged (21,000*g* at 4°C for 30 minutes), and the supernatant was discarded. The pellet was then homogenized in 40 μL of TFA extraction buffer (1% TFA, 5 mM TCEP, 5 mM L-methionine) using a handheld Teflon homogenizer. The homogenate was centrifuged (21,000*g* at 4°C for 30 minutes), and the resulting supernatant was desalted with 5 volumes of MPA (0.1% formic acid in HPLC grade water) using a 10 kDa molecular weight cutoff filter (Amicon). Protein normalization was performed using a bovine serum standard curve and Bradford assay.

A NanoAcquity LC system (Waters) was used as part of the reverse phase chromatography (RPC) system. In total, 500 ng of the protein extracts was run through a home-packed PLRP-S capillary column (200 mm long, 0.25 mm Inner diameter (i.d.), 5 μm particle size, 1,000 Å pore size; Agilent Technology). The column was heated to 60°C at an 8 μL/min flow rate. The gradient is as follows, in terms of MPB (0.1% formic acid in 50:50 acetonitrile/ethanol): 10% MPB at 0–5 minutes, slow increase to 65% at 5–65 minutes, 95% at 70–75 minutes, back to 10% at 75.1 minutes, and steadily at 10% until 80 minutes. The eluted proteins were analyzed using a Bruker Impact II Q-TOF mass spectrometer (Bruker). The mass spectrometer parameters were as follows: end plate offset,500 V; capillary voltage, 4500 V; nebulizer, 0.3 bar; dry gas flow rate, 4.0 L/min at 200°C; quadrupole low mass, 650 *m/z*; scan rate, 1 Hz; *m/z* range, 200–3,000 *m/z*. Three technical replicates were collected for 1 sample across instrument run time to ensure data reproducibility and stability.

DataAnalysis 4.3 software (Bruker Daltonics) was used for all MS data analysis. A smoothing width of 2.01 using the Gaussian algorithm was applied to each chromatogram. The Maximum Entropy algorithm within the DataAnalysis 4.3 software was implemented to deconvolute spectra for proteins of interest at resolving power of 50,000. The sophisticated numerical annotation procedure (SNAP) algorithm was used to determine the monoisotopic masses of all deconvoluted ions. Relative quantification of protein phosphorylation is reported from deconvoluted spectra, in a relative abundance of a particular proteoform (Ptotal). Ptotal is equivalent to the ratio of the peak intensity of the proteoform (mol Pi) to the total sum of peak intensities of all proteoforms (mol protein) of the same protein. Statistical significances were determined by normal 2-tailed *t* test with α = 0.05 with 2-sided analysis. All data represented as data mean ± SEM.

### Statistics.

All statistical analyses were performed as 2-tailed Student’s *t* tests with assumed normal distribution, unless otherwise stated, with α = 0.05. Thus, a *P* value less than 0.05 was considered significant. All data are represented as data mean ± SEM, unless otherwise stated.

### Study approval.

Protocols for the generation of hiPSCs were approved by the Stanford University Human Subjects Research IRB, and written consent was obtained from all study participants.

### Data availability.

Source data for this manuscript available via MassIVE repository at massive.ucsd.edu with identifier: MassIVE MSV000091869. All raw data values are reported in the Supporting Data Values file. Any additional information is available by request from the corresponding authors.

## Author contributions

KJR, TJK, JCR, WJDL, ETF, and YG contributed to study design. KJR cultured cells upon differentiation, generated hiPSC-ECTs, performed proteomics sample preparation, analyzed targeted proteomics data, prepared hiPSC-ECTs for sections, imaged IHC images, processed data, and wrote the manuscript. WJDL gathered and analyzed all functional data on hiPSC-ECTs. MWM, KJR, and TJA analyzed global proteomics data. JAM and EFB assisted with collection of targeted proteomics data. JZ and GK differentiated cells into CMs and CFs. YZ assisted with proteomics data collection. TJK, JCR, and YG edited the manuscript and provided supervision of the study. All authors contributed to the writing of the manuscript.

## Supplementary Material

Supplemental data

Supplemental data set 1

Supplemental data set 2

Supporting data values

## Version 1 11/21/2023

In-Press Preview

## Version 2 01/09/2024

Electronic publication

## References

[1] Kamp TJ, Lyons GE (2009). On the road to iPS cell cardiovascular applications. Circ Res.

[2] Zhang J (2009). Functional cardiomyocytes derived from human induced pluripotent stem cells. Circ Res.

[3] Davis J (2021). In vitro model of ischemic heart failure using human induced pluripotent stem cell-derived cardiomyocytes. JCI Insight.

[4] Lan F (2013). Abnormal calcium handling properties underlie familial hypertrophic cardiomyopathy pathology in patient-specific induced pluripotent stem cells. Cell Stem Cell.

[5] Novak A (2015). Functional abnormalities in iPSC-derived cardiomyocytes generated from CPVT1 and CPVT2 patients carrying ryanodine or calsequestrin mutations. J Cell Mol Med.

[6] Yazawa M (2011). Using induced pluripotent stem cells to investigate cardiac phenotypes in Timothy syndrome. Nature.

[7] Ahmed RE (2020). A brief review of current maturation methods for human induced pluripotent stem cells-derived cardiomyocytes. Front Cell Dev Biol.

[8] Bedada FB (2016). Maturation status of sarcomere structure and function in human iPSC-derived cardiac myocytes. Biochim Biophys Acta.

[9] Ebert A (2019). Proteasome-dependent regulation of distinct metabolic states during long-term culture of human iPSC-derived cardiomyocytes. Circ Res.

[10] Parikh SS (2017). Thyroid and glucocorticoid hormones promote functional T-tubule development in human-induced pluripotent stem cell-derived cardiomyocytes. Circ Res.

[11] Hirt MN (2014). Functional improvement and maturation of rat and human engineered heart tissue by chronic electrical stimulation. J Mol Cell Cardiol.

[12] De Lange WJ (2021). Human iPSC-engineered cardiac tissue platform faithfully models important cardiac physiology. Am J Physiol Heart Circ Physiol.

[13] Cai W (2019). An unbiased proteomics method to assess the maturation of human pluripotent stem cell-derived cardiomyocytes. Circ Res.

[14] Campostrini G (2021). Generation, functional analysis and applications of isogenic three-dimensional self-aggregating cardiac microtissues from human pluripotent stem cells. Nat Protoc.

[15] Hinson JT (2015). Heart disease. Titin mutations in iPS cells define sarcomere insufficiency as a cause of dilated cardiomyopathy. Science.

[16] Burridge PW (2014). Chemically defined generation of human cardiomyocytes. Nat Methods.

[17] https://www.miltenyibiotec.com/_Resources/Persistent/90983e1c538c4f44db385930feb5c42aba62f193/App_note_PSC_derived_cardiomyocytes.pdf.

[18] Tohyama S (2013). Distinct metabolic flow enables large-scale purification of mouse and human pluripotent stem cell-derived cardiomyocytes. Cell Stem Cell.

[19] Shiba Y (2016). Allogeneic transplantation of iPS cell-derived cardiomyocytes regenerates primate hearts. Nature.

[20] Kawamura M (2017). Enhanced therapeutic effects of human iPS cell derived-cardiomyocyte by combined cell-sheets with omental flap technique in porcine ischemic cardiomyopathy model. Sci Rep.

[21] Serpooshan V (2017). Bioacoustic-enabled patterning of human iPSC-derived cardiomyocytes into 3D cardiac tissue. Biomaterials.

[22] Miklas JW (2019). TFPa/HADHA is required for fatty acid beta-oxidation and cardiolipin re-modeling in human cardiomyocytes. Nat Commun.

[23] Sharma A (2014). Human induced pluripotent stem cell-derived cardiomyocytes as an in vitro model for coxsackievirus B3-induced myocarditis and antiviral drug screening platform. Circ Res.

[24] Cohn R (2019). A contraction stress model of hypertrophic cardiomyopathy due to sarcomere mutations. Stem Cell Reports.

[25] Kallepitis C (2017). Quantitative volumetric Raman imaging of three dimensional cell cultures. Nat Commun.

[26] Arvanitis M (2020). Genome-wide association and multi-omic analyses reveal ACTN2 as a gene linked to heart failure. Nat Commun.

[27] Sharma A (2018). Use of human induced pluripotent stem cell-derived cardiomyocytes to assess drug cardiotoxicity. Nat Protoc.

[28] Martin RM (2020). Improving the safety of human pluripotent stem cell therapies using genome-edited orthogonal safeguards. Nat Commun.

[29] Kalmykov A (2019). Organ-on-e-chip: three-dimensional self-rolled biosensor array for electrical interrogations of human electrogenic spheroids. Sci Adv.

[30] Correia C (2017). Distinct carbon sources affect structural and functional maturation of cardiomyocytes derived from human pluripotent stem cells. Sci Rep.

[31] Gao L (2020). Exosomes secreted by hiPSC-derived cardiac cells improve recovery from myocardial infarction in swine. Sci Transl Med.

[32] Boon R (2020). Amino acid levels determine metabolism and CYP450 function of hepatocytes and hepatoma cell lines. Nat Commun.

[33] Friedman CE (2018). Single-cell transcriptomic analysis of cardiac differentiation from human PSCs reveals HOPX-dependent cardiomyocyte maturation. Cell Stem Cell.

[34] Long C (2018). Correction of diverse muscular dystrophy mutations in human engineered heart muscle by single-site genome editing. Sci Adv.

[35] Foinquinos A (2020). Preclinical development of a miR-132 inhibitor for heart failure treatment. Nat Commun.

[36] Sharma A (2017). High-throughput screening of tyrosine kinase inhibitor cardiotoxicity with human induced pluripotent stem cells. Sci Transl Med.

[37] Park SJ (2019). Dual stem cell therapy synergistically improves cardiac function and vascular regeneration following myocardial infarction. Nat Commun.

[38] Shadrin IY, A al (2017). Cardiopatch platform enables maturation and scale-up of human pluripotent stem cell-derived engineered heart tissues. Nat Commun.

[39] Tiburcy M (2017). Defined engineered human myocardium with advanced maturation for applications in heart failure modeling and repair. Circulation.

[40] Chai RJ (2021). Disrupting the LINC complex by AAV mediated gene transduction prevents progression of Lamin induced cardiomyopathy. Nat Commun.

[41] Smith LM, Kelleher NL (2018). Proteoforms as the next proteomics currency. Science.

[42] Lian X (2013). Directed cardiomyocyte differentiation from human pluripotent stem cells by modulating Wnt/β-catenin signaling under fully defined conditions. Nat Protoc.

[43] Brown KA (2019). A photocleavable surfactant for top-down proteomics. Nat Methods.

[44] Bayne EF (2021). Multiomics method enabled by sequential metabolomics and proteomics for human pluripotent stem-cell-derived cardiomyocytes. J Proteome Res.

[45] Kanehisa M, Goto S (2000). KEGG: kyoto encyclopedia of genes and genomes. Nucleic Acids Res.

[46] UniProt Consortium (2023). UniProt: the universal protein knowledgebase in 2023. Nucleic Acids Res.

[47] Thomas PD (2022). PANTHER: making genome-scale phylogenetics accessible to all. Protein Sci.

[48] Zhang J (2019). Functional cardiac fibroblasts derived from human pluripotent stem cells via second heart field progenitors. Nat Commun.

[49] Linke WA (2008). Sense and stretchability: the role of titin and titin-associated proteins in myocardial stress-sensing and mechanical dysfunction. Cardiovasc Res.

[50] Samarel AM (2005). Costameres, focal adhesions, and cardiomyocyte mechanotransduction. Am J Physiol Heart Circ Physiol.

[51] Melby JA (2021). Functionally integrated top-down proteomics for standardized assessment of human induced pluripotent stem cell-derived engineered cardiac tissues. J Proteome Res.

[52] Cai W (2018). Temperature-sensitive sarcomeric protein post-translational modifications revealed by top-down proteomics. J Mol Cell Cardiol.

[53] Chait BT (2006). Chemistry. Mass spectrometry: bottom-up or top-down?. Science.

[54] Kobayashi T (2005). Calcium, thin filaments, and the integrative biology of cardiac contractility. Annu Rev Physiol.

[55] Kajioka S (2012). Endogenous cardiac troponin T modulates Ca(2+)-mediated smooth muscle contraction. Sci Rep.

[56] Aballo TJ (2023). Integrated proteomics reveals alterations in sarcomere composition and developmental processes during postnatal swine heart development. J Mol Cell Cardiol.

[57] Sitbon YH (2020). Insights into myosin regulatory and essential light chains: a focus on their roles in cardiac and skeletal muscle function, development and disease. J Muscle Res Cell Motil.

[58] Rupert CE (2020). Practical adoption of state-of-the-art hiPSC-cardiomyocyte differentiation techniques. PLoS One.

[59] De Lange WJ (2023). cMyBP-C ablation in human engineered cardiac tissue causes progressive Ca2+-handling abnormalities. J Gen Physiol.

[60] Aït Mou Y (2009). Late exercise training improves non-uniformity of transmural myocardial function in rats with ischaemic heart failure. Cardiovasc Res.

[61] Gianni D (2005). SERCA2a in heart failure: role and therapeutic prospects. J Bioenerg Biomembr.

[62] Chu G, Kranias EG (2006). Phospholamban as a therapeutic modality in heart failure. Novartis Found Symp.

[63] Lodrini AM, Goumans MJ (2021). Cardiomyocytes cellular phenotypes after myocardial infarction. Front Cardiovasc Med.

[64] Fenix AM (2018). Muscle-specific stress fibers give rise to sarcomeres in cardiomyocytes. Elife.

[65] Dispersyn GD (2002). Dissociation of cardiomyocyte apoptosis and dedifferentiation in infarct border zones. Eur Heart J.

[66] Melby JA (2023). High sensitivity top-down proteomics captures single muscle cell heterogeneity in large proteoforms. Proc Natl Acad Sci U S A.

[67] Bayne EF (2023). Top-down proteomics of myosin light chain isoforms define chamber-specific expression in the human heart. J Mol Cell Cardiol.

[68] Minor AJ, Coulombe KLK (2022). Stimulating calcium handling in hiPSC-derived engineered cardiac tissues enhances force production. Stem Cells Transl Med.

[69] Zhao Y (2019). A platform for generation of chamber-specific cardiac tissues and disease modeling. Cell.

[70] De Lange WJ (2011). Neonatal mouse-derived engineered cardiac tissue: a novel model system for studying genetic heart disease. Circ Res.

[71] Aballo TJ (2021). Ultrafast and reproducible proteomics from small amounts of heart tissue enabled by Azo and timsTOF pro. J Proteome Res.

[72] Mann M (2021). Discovery of RSV-induced BRD4 protein interactions using native immunoprecipitation and parallel accumulation-serial fragmentation (PASEF) mass spectrometry. Viruses.

[73] Demichev V (2020). DIA-NN: neural networks and interference correction enable deep proteome coverage in high throughput. Nat Methods.

[74] Wieczorek S (2017). DAPAR & ProStaR: software to perform statistical analyses in quantitative discovery proteomics. Bioinformatics.

