# Supplementary material for: Lactate- and immunomagnetic-purified hiPSC–derived cardiomyocytes generate comparable engineered cardiac tissue constructs
Source: JCI Insight. 2024 Jan 9;9(1):e172168. doi: 10.1172/jci.insight.172168 (PMC10906451; doi:10.1172/jci.insight.172168)
Supplement: Supplemental data [file jciinsight-9-172168-s154.pdf]

Supplemental Information

Lactate and Immunomagnetic-purified hiPSC-derived Cardiomyocytes Generate Comparable Engineered Cardiac Tissue Constructs

Kalina J. Rossler[1,2], Willem J. de Lange[3], Morgan W. Mann[4], Timothy J. Aballo[1,2], Jake A. Melby[5], Jianhua Zhang[4], Gina Kim[4], Elizabeth F. Bayne[5], Yanlong Zhu[6], Emily T. Farrell[3], Timothy J. Kamp[2,4*], J. Carter Ralphe[3*], and Ying Ge[2,5,6*]

[1]Molecular and Cellular Pharmacology Training Program, University of Wisconsin-Madison, Madison, WI 53705, USA

[2]Department of Cell and Regenerative Biology, University of Wisconsin-Madison, Madison, WI, 53705, USA

[3]Department of Pediatrics, University of Wisconsin-Madison Madison, WI 53705, USA

[4]Department of Medicine, University of Wisconsin-Madison Madison, WI 53705, USA

[5]Department of Chemistry, University of Wisconsin-Madison, Madison, WI 53706, USA

[6]Human Proteomics Program, School of Medicine and Public Health, University of Wisconsin-Madison, Madison, WI, 53705, USA

*To whom correspondence should be addressed: Ying Ge, ying.ge@wisc.edu; Timothy J. Kamp, tjk@medicine.wisc.edu; J. Carter Ralphe, jcralphe@pediatrics.wisc.edu 1111 Highland Ave., WIMR II 8551, Madison, WI 53705.

Keywords: stem cell; cardiac modeling; cardiomyocyte purification; engineered cardiac tissue; hiPSC-CM

SUPPLEMENTAL TABLE OF CONTENTS

Supplemental Table and Figures ……………………………………………..……..Page #

Table 1. Extraction Ion Chromatograms for Top-down Proteomics……………………..…3

Supplemental Figure 1. PANTHER Pathway Analysis of Differentially Expressed Genes for Lactate hiPSC-CMs……………………………………………………………………. 4

Supplemental Figure 2. Immunohistochemistry Images for Sarcomere Length Analysis……………………………………………………………………………………..5

Supplemental Figure 3. Overview of Functional Parameters………………………………6

Supplemental Figure 4. Representative Automaticity Traces of MACS and Lactate hiPSC-ECTs…………………………………………………………………………………..7

Supplemental Figure 5. SDS-Page Gel for Reproducibility of Proteomics Extraction…...8

Supplemental Figure 6. Total Ion Chromatograms (TICs) for Global Proteomics …..... 9

Supplemental Figure 7. Differentially Expressed Proteins between Lactate and MACS hiPSC-ECTs…………………………………………………………………………..……..10

Supplemental Figure 8. Pathway Analysis of hiPSC-ECT proteome………………..…..11

Supplemental Figure 9. Base Peak Chromatograms (BPCs) of Intact Sarcomere Proteomics…………………………………………………………………………………...12

Supplemental Figure 10. Instrument Linear Response for Intact Protein Analysis……………………………………………………………………………………13

Supplemental Figure 11. Instrument Stability using Base Peak Chromatograms …………………………………………………………………………………………...…14

Supplemental Figure 12. Spectra and isotopic resolution for cα-actin …………………………………………………………………………………………...…15

Protein Name	Ions for Extracted Ion Chromatograms (EICs)
cTnT	749.8962; 787.4432; 844.3806; 874.7710
ssTnI	744.5066; 771.0607; 799.5637; 830.2574
α-Tpm	764.4842; 801.7475; 821.7771
MLC-1v	780.9363; 810.0058; 841.0839; 874.7648
MLC-1a	826.9432; 859.9819; 895.8552; 934.6952
MLC-2v	814.0454; 850.9555; 891.4288; 935.9504
cα-actin	837.4561; 854.8404; 872.7114; 891.1384
TnC	1025.7064; 1085.8935; 1153.6982

Table 1. Ions used for quantitative EICs for top-down proteomics.

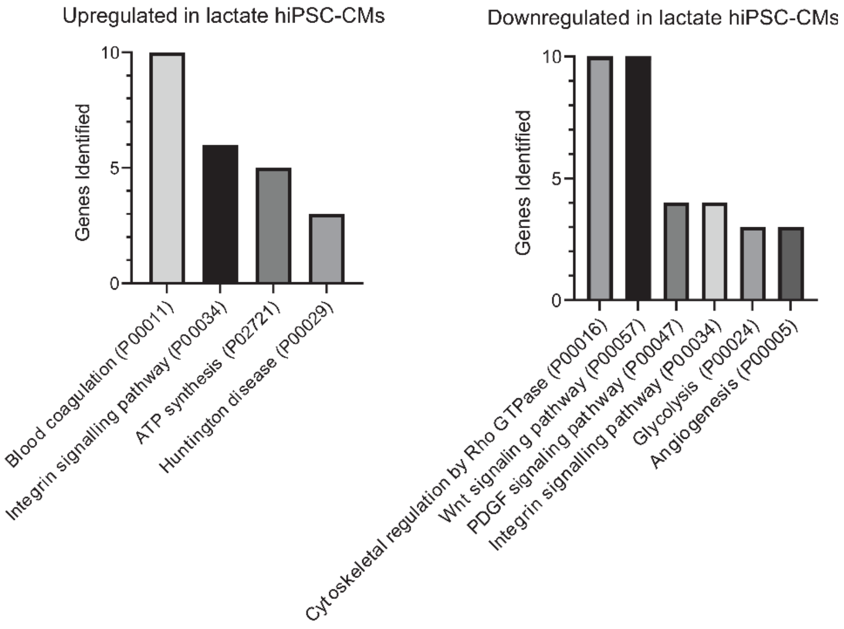

Supplemental Figure 1. PANTHER Pathway Analysis of Differentially Expressed Genes for Lactate hiPSC-CMs. (A) Top four pathways with gene ontology (GO) identifier for upregulated pathways in lactate hiPSC-CMs. (B) Top six pathways with GO identifier for downregulated pathways in lactate hiPSC-CMs. Pathways identified using differentially expressed proteins with greater than 2 proteins per pathway.

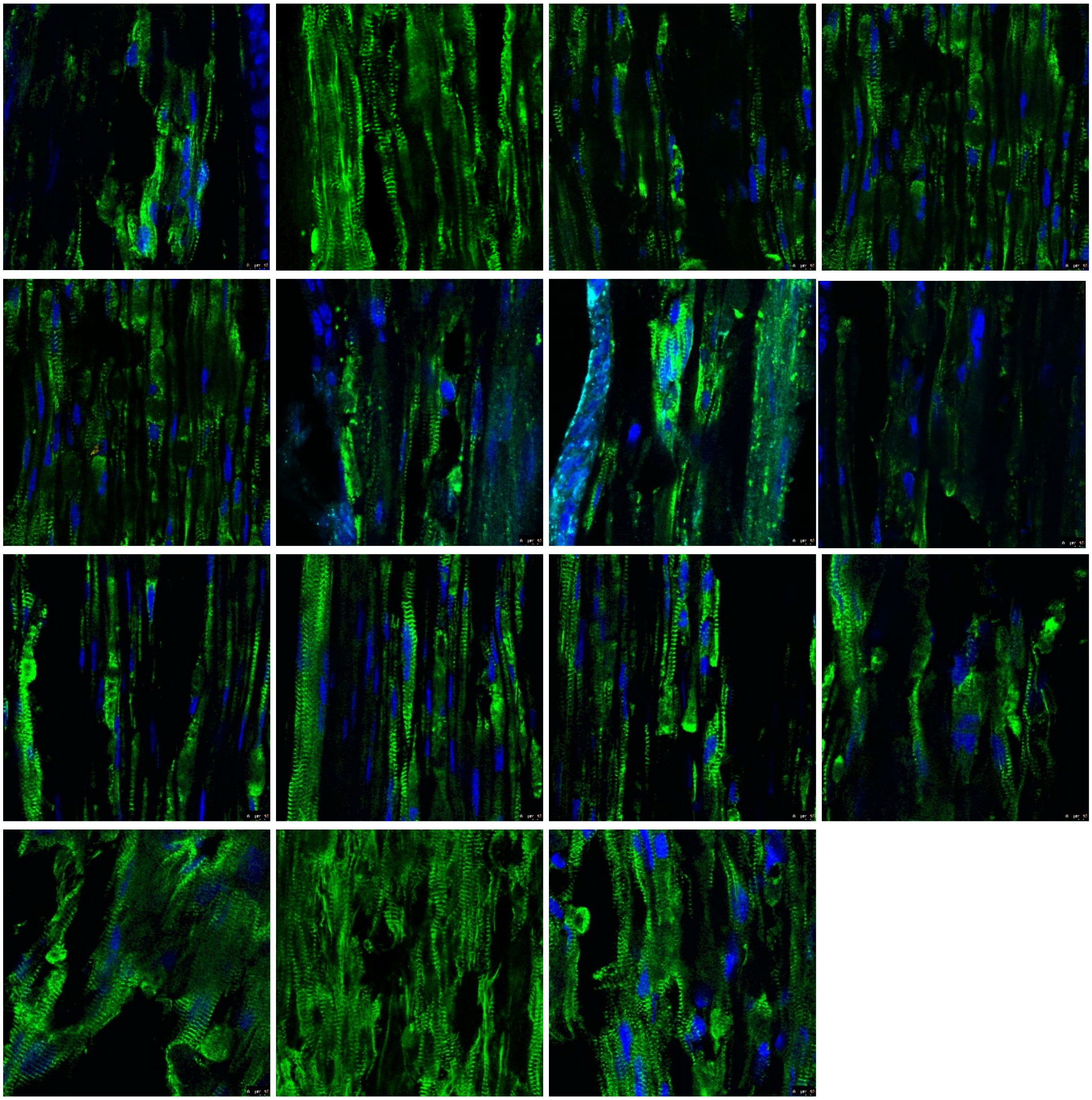

Supplemental Figure 2. Immunohistochemistry Images for Sarcomere Length Analysis. *Top (left to right) to Bottom – Condition Replicate Number (Slide Number):* Lactate 1(1), Lactate 2(2), Lactate 2(4), Lactate 2(6), Lactate 2(7), Lactate 3(11), Lactate 3(16), Lactate 3(18), MACS 1(1), MACS 1(4), MACS 1(5), MACS 2(1), MACS 2(2), MACS 3(1), MACS 3(2). Green fluorescence indicates alpha-actinin staining. DAPI is indicated in blue. Immunohistochemistry was performed with hiPSC-ECTs at day 58, with day 0 being the generation of hiPSC-ECTs.

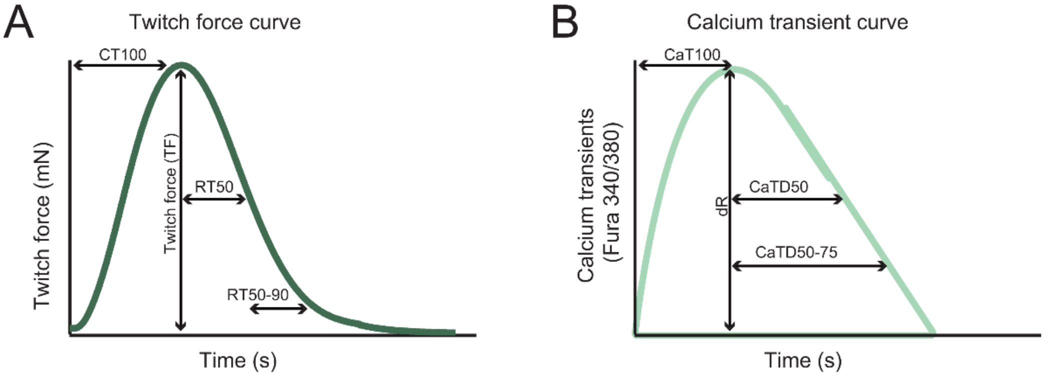

Supplemental Figure 3. Overview of Functional Parameters. (A) Twitch force parameters as indicated: twitch force amplitude (TF), time from pacing stimulus to twitch force peak (CT100), time from twitch force peak to 50% twitch force decay (RT50), and time from 50% to 90% twitch force decay (RT50–90). (B) Ca^{2+} transient parameters as indicated: Ca^{2+}TR peak (dR), time to Ca^{2+}TR peak (CaT100) from Ca^{2+}TR peak to 50% Ca^{2+}TR decay (CaDT50), and time from 50% Ca^{2+}TR decay to 75% Ca^{2+}TR decay (CaDT50-75).

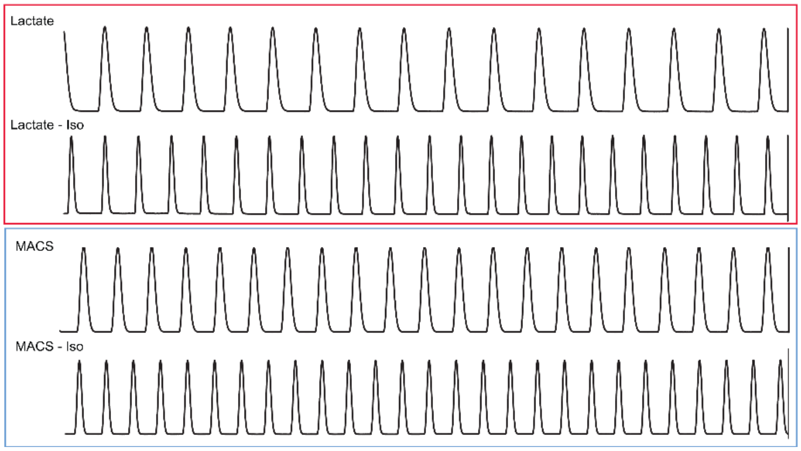

Supplemental Figure 4. Representative Automaticity Traces of MACS and Lactate hiPSC-ECTs. Traces representative of automaticity in lactate (red) and MACS (blue) hiPSC-ECTs for approximately 15 seconds after functional evaluation. Traces indicate no arrythmias in automaticity pre- or post-treatment with 1 μM isoproterenol. Functional testing was performed with hiPSC-ECTs at day 58, with day 0 being the generation of hiPSC-ECTs.

‘

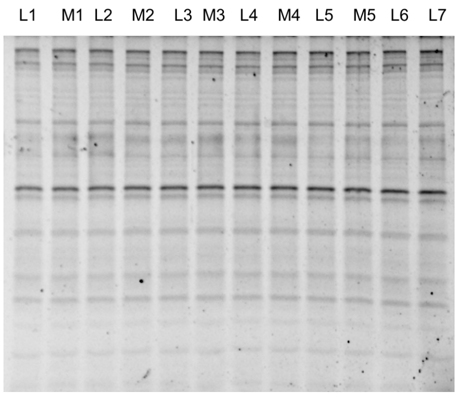

Supplemental Figure 5. SDS-Page Gel for Reproducibility of Proteomics Extraction. 12.5% SDS-Page gel with lactate (L#) and MACS (M#) replicates. 500 ng of protein lysate was loaded onto the gel.

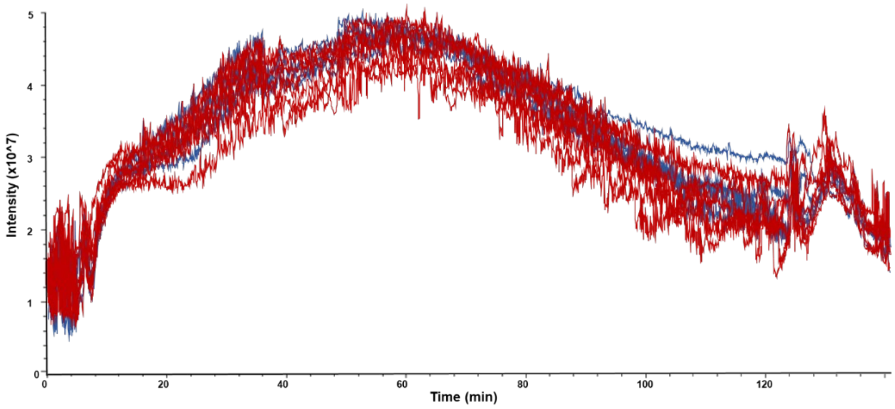

Supplemental Figure 6. Total Ion Chromatograms (TICs) for Global Proteomics. TIC traces are shown for lactate (red) and MACS (blue) biological replicates. Overlay of the TICs indicates good reproducibility for all samples.

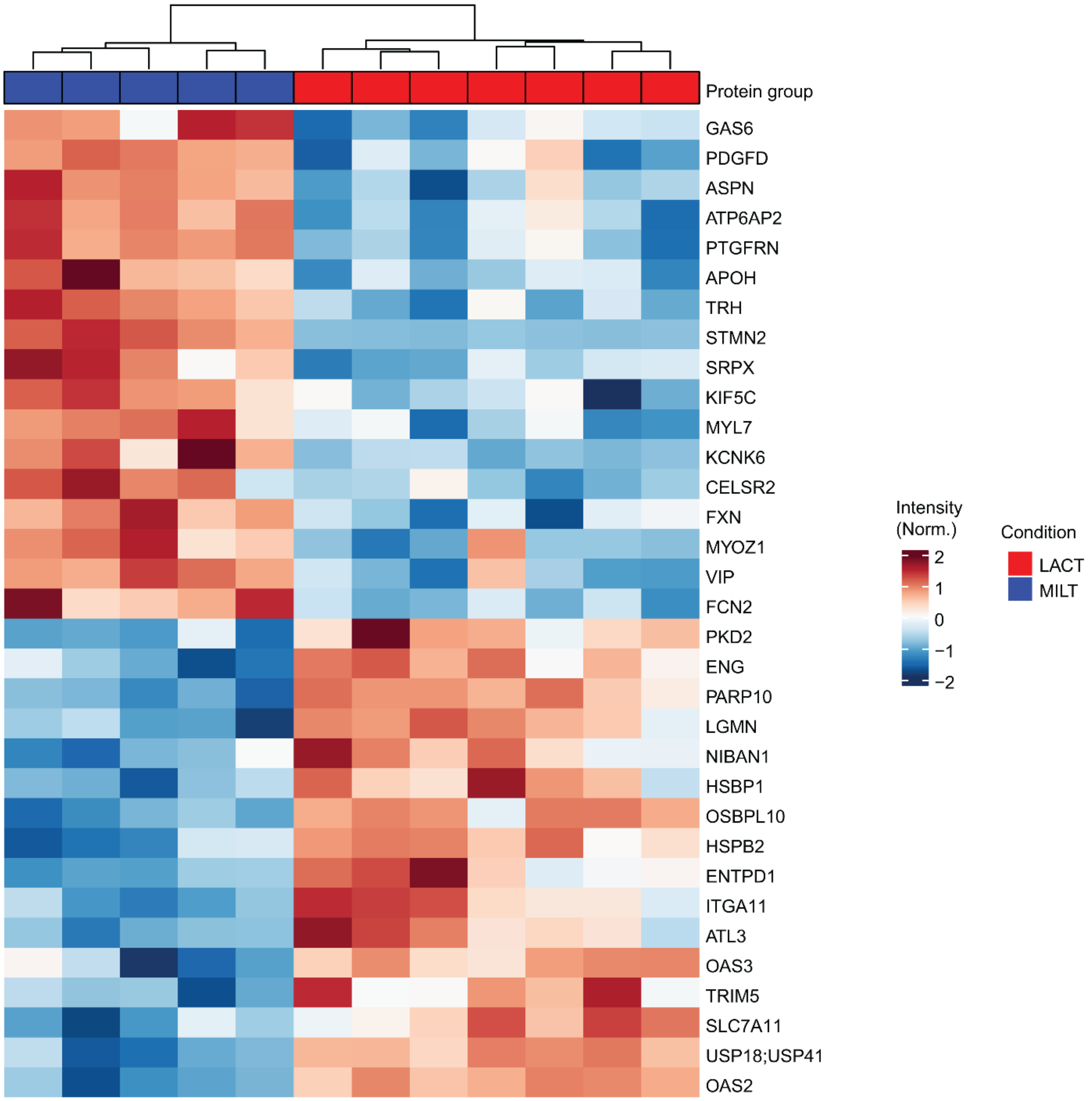

Supplemental Figure 7. Differentially Expressed Proteins between Lactate and MACS hiPSC-ECTs. Heatmap depicts normalized intensity values between differentially expressed proteins of day 58 lactate (LACT) and MACS (MILT) hiPSC-ECTs.

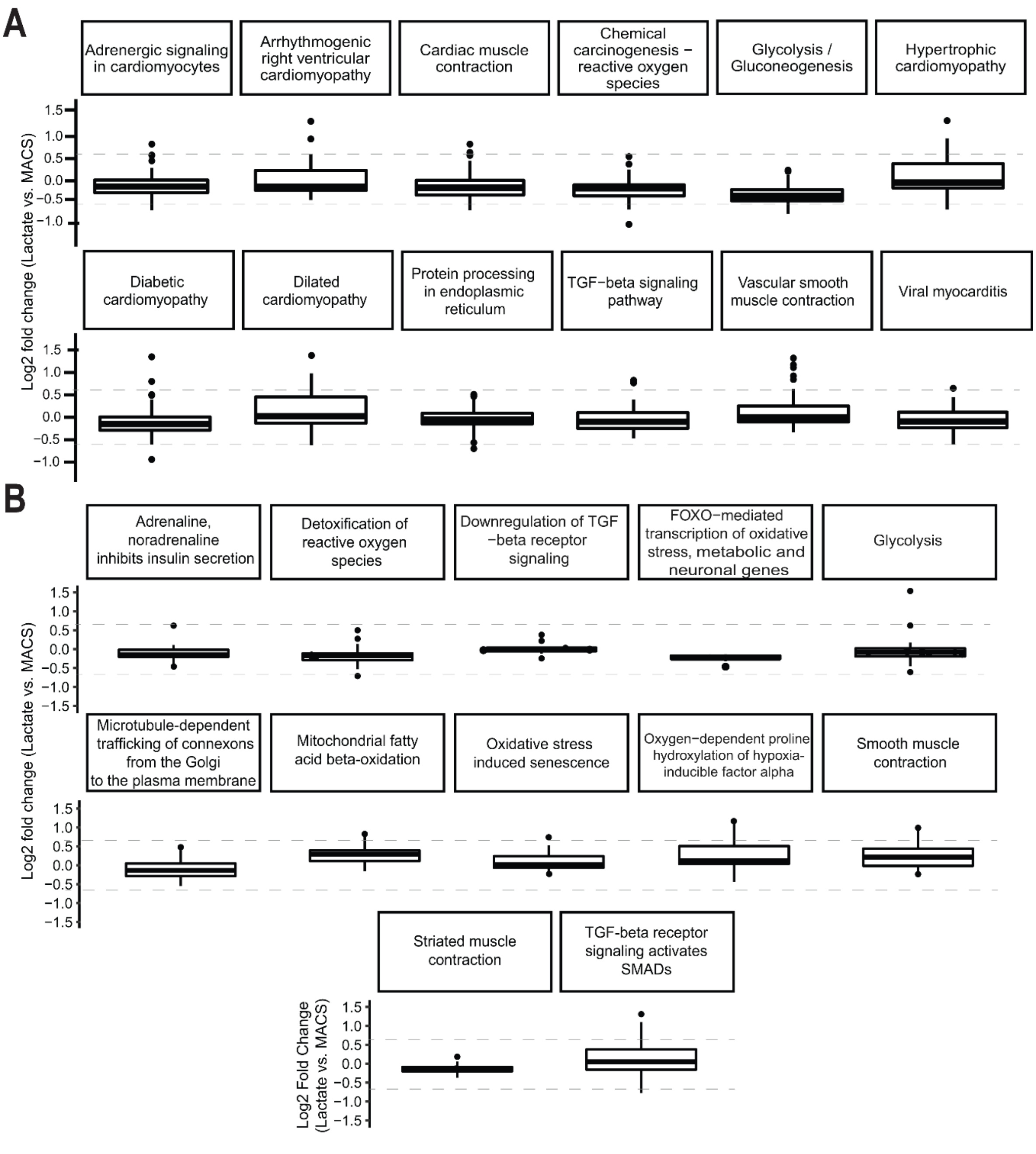

Supplemental Figure 8. Pathway Analysis of hiPSC-ECT proteome. (A) KEGG pathway analysis for global protein expression of day 58 lactate and MACS hiPSC-ECTs. (B) UniProt pathway analysis for global protein expression of day 58 lactate and MACS hiPSC-ECTs. Cut-off values of 0.6 log_2 fold change indicated by dotted gray line.

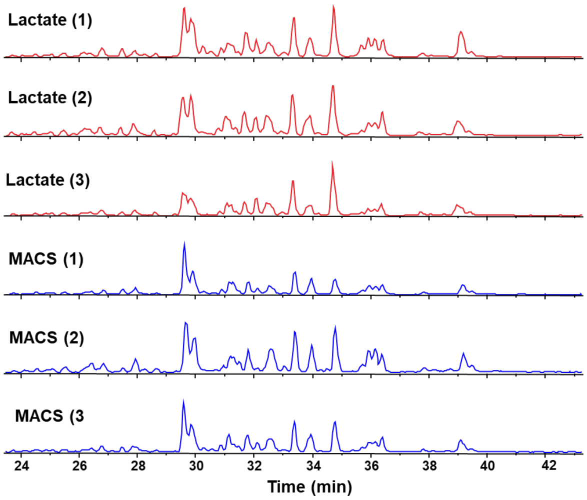

Supplemental Figure 9. Base Peak Chromatograms (BPCs) of Intact Sarcomere Proteomics. BPC traces are shown for representative lactate (red) and MACS (blue) biological replicates with normalized intensity. Similar intensity and BPC shape indicates good reproducibility for all samples.

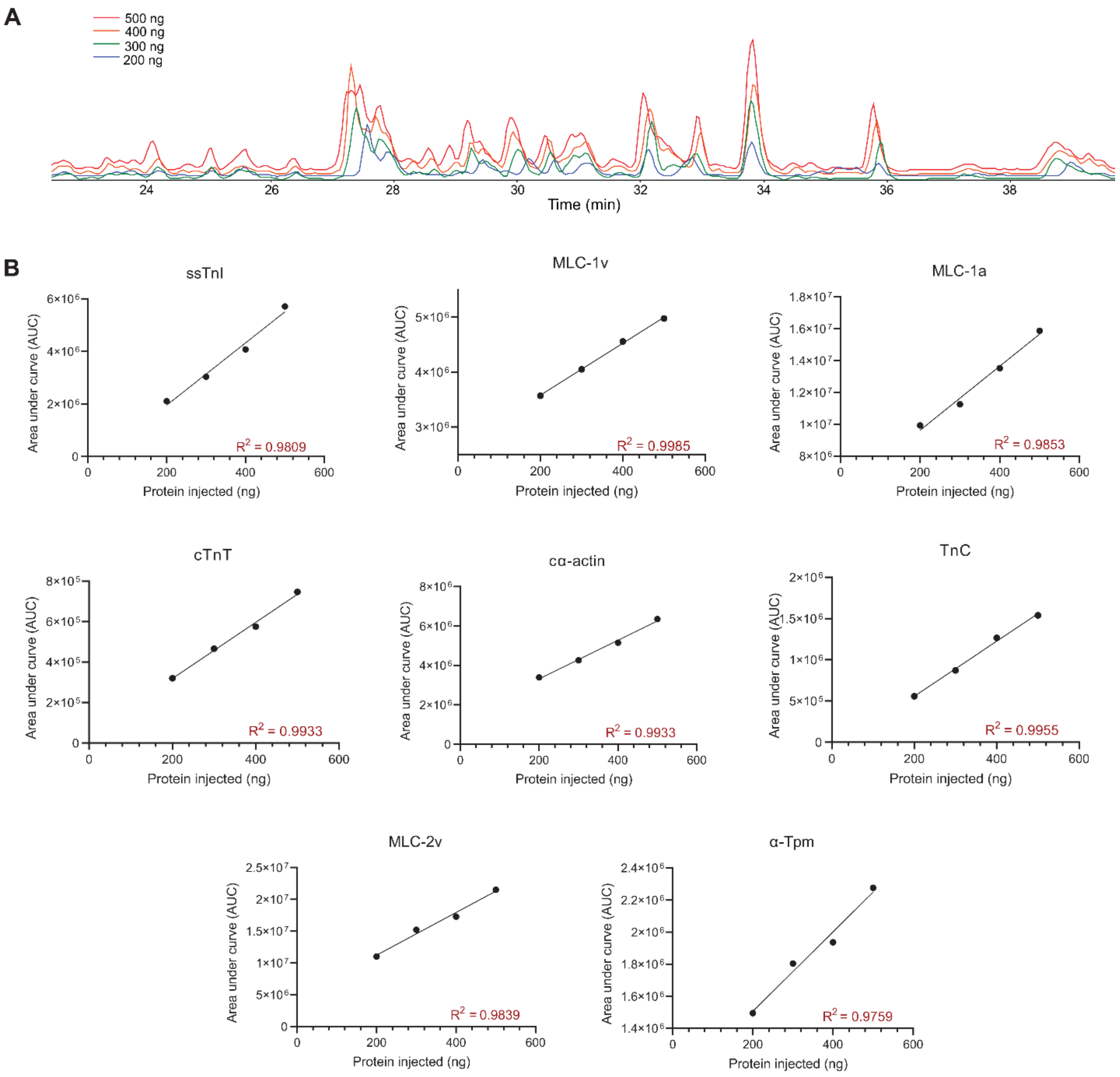

Supplemental Figure 10. Instrument Linear Response for Intact Protein Analysis. A) Various protein amounts injected on the IMPACT II to establish linear response curve – represented by base peak chromatogram. Trace color (total protein injected); Red (500 ng), Orange (400 ng), Green (300 ng), Blue (200 ng). B) Various linear curves generated for proteins identified. R^2 values determined by simple linear regression.

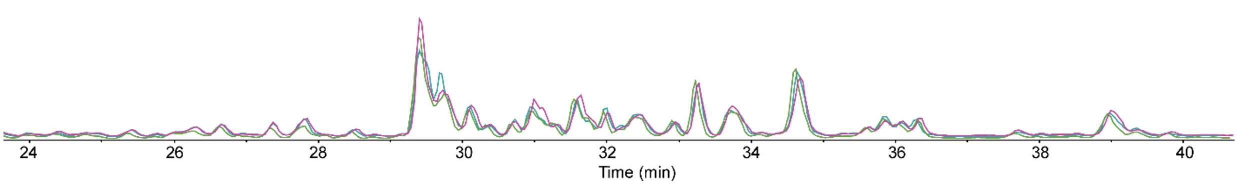

Supplemental Figure 11. Instrument Stability using Base Peak Chromatograms. Injections from example lactate injection shown from beginning (purple), middle (green), and end (blue) of instrument runs.

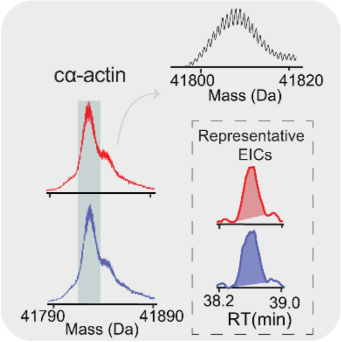

Supplemental Figure 12. Spectra and isotopic resolution for cα-actin. Spectra and isotopic resolution given for cardiac alpha-actin (cα-actin) with retention times (RT) shown with extraction ion chromatograms (EICs).
