# Supplementary material for: Lactate- and immunomagnetic-purified hiPSC–derived cardiomyocytes generate comparable engineered cardiac tissue constructs
Source: JCI Insight. 2024 Jan 9;9(1):e172168. doi: 10.1172/jci.insight.172168 (PMC10906451; doi:10.1172/jci.insight.172168)
Supplement: Supplemental data set 1 [file jciinsight-9-172168-s155.pdf]

Mycoplasma Assay Report

PCR-based assay performed by WiCell

Kalina Rossler, UW Madison

10May22

Form SOP-83.01
Version 5.0

Sample Name	Result	Interpretation
SCVI 8 (91931)	Negative	Band was not seen at 270bp, indicating the absence of mycoplasma.
Positive (+) Control	Positive	
Negative (-) Control	Negative	

Assay Description
Sample is tested for presence of mycoplasma using EZ-PCR™ Mycoplasma Detection Kit (Sartorius).

5/10/2022	5/10/2022	5/12/2022
X Justin Hobson	X Kayla Janke	X Dawn Graham
Tech #1<br>Characterization<br>Signed by: Hobson, Justin	Tech #2<br>Characterization<br>Signed by: Janke, Kayla	QA Review<br>Quality Assurance<br>Signed by: Graham, Dawn

Unless otherwise mutually agreed in writing, the services provided to you hereunder by WiCell Research Institute, Inc. ("WiCell") are governed solely by WiCell's Terms and Conditions of Service, found at www.wicell.org/privacyandterms. Any terms you may attach to a purchase order or other document that are inconsistent, add to, or conflict with WiCell's Terms and Conditions of Service are null and void and of no legal force or effect.

A gel image is available upon request.
