# Supplementary material for: Lactate- and immunomagnetic-purified hiPSC–derived cardiomyocytes generate comparable engineered cardiac tissue constructs
Source: JCI Insight. 2024 Jan 9;9(1):e172168. doi: 10.1172/jci.insight.172168 (PMC10906451; doi:10.1172/jci.insight.172168)
Supplement: Supplemental data set 2 [file jciinsight-9-172168-s156.pdf]

R Notebook

Code

Hide

```
library(tcltk)
exFile <- tk_choose.files()
```

#sample data setup

Hide

```
library(DAPAR)
```

```
Registered S3 method overwritten by 'htmlwidgets':
  method           from
  print.htmlwidget tools:rstudio
Registered S3 method overwritten by 'quantmod':
  method            from
  as.zoo.data.frame zoo
Registered S3 method overwritten by 'data.table':
  method           from
  print.data.table

This is the 'DAPAR' version 1.28.5.

  To get started, visit
    http://www.prostar-proteomics.org/


Warning messages:
1: R graphics engine version 15 is not supported by this version of RStudio. The Plots tab will
be disabled until a newer version of RStudio is installed.
2: In fun(libname, pkgname) :
  mzR has been built against a different Rcpp version (1.0.8.3)
than is installed on your system (1.0.9). This might lead to errors
when loading mzR. If you encounter such issues, please send a report,
including the output of sessionInfo() to the Bioc support forum at
https://support.bioconductor.org/. For details see also
https://github.com/sneumann/mzR/wiki/mzR-Rcpp-compiler-linker-issue.
```

Hide

```
dir.create("MSnSET-DIA/")
```

```
Warning in dir.create("MSnSET-DIA/") : 'MSnSET-DIA' already exists
```

Hide

```
exData <- read.table(exFile, sep="\t", header=TRUE, quote="")
#expIndex <- c(6:8, 12:14, 9:11, 15:17)
expIndex <- c(6:17)

#this is setting up the sample table

Condition <- c(rep("LACT", 7), rep("MILT", 5))
Sample.name <- c(paste("LACT", seq(1:7), sep="_"), paste("MILT", seq(1:5), sep="_"))

colnames(exData)[expIndex] <- Sample.name
write.table(exData, file="MSnSET-DIA/exFile.tsv", row.names=FALSE, sep="\t")
exFile2 <- "MSnSET-DIA/exFile.tsv"

metaData <- data.frame(Sample.name, Condition)

prot <- createMSnset(file = exFile2, metadata = metaData,
                     indExpData = expIndex,
                     logData = TRUE,
                     replaceZeros = TRUE,
                     pep_prot_data = "protein",
                     colnameForID = "Protein.Group")
saveRDS(prot, file="MSnSET-DIA/hSAEC_RSV_ZL_GBUP_NOV2021.msnset")
```

#filtering

Hide

```
metacell.mask <- match.metacell(GetMetacell(prot), 'missing',
                                level="protein")

#this is data specific - build this command

conditionIndices <- GetIndices_BasedOnConditions(
  metacell.mask, type = "AtLeastOneCond",
  Biobase::pData(prot)$Condition, op = '<=',
  th=2, percent = .4
  )

#apply your built command to your data

prot.Filt1 <- MetaCellFiltering(prot, conditionIndices, "keep")[[1]]

saveRDS(prot.Filt1, file="MSnSET-DIA/hSAEC_RSV_ZL_GBUP_NOV2021.filt.msnset")
```

#this is a set of diagnostic plots - shows you what normalization is doing

Hide

```
legend <- Biobase::pData(prot.Filt1)[,"Condition"]
violinPlotD(prot.Filt1, legend=legend, conds = legend)
```

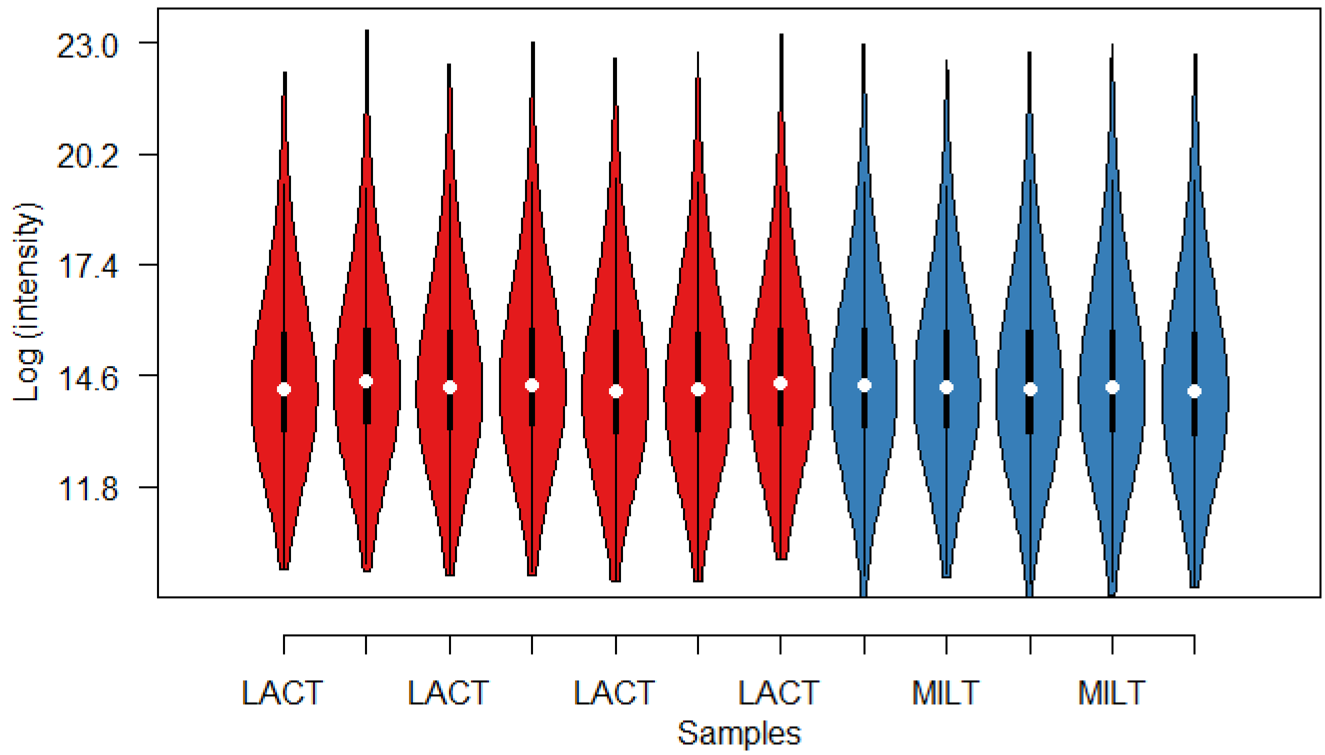

Hide

```
prot.Norm <- wrapper.normalizeD(prot.Filt1, method = "QuantileCentering",
                                type="overall", conds=legend, quantile=.15)
#Below is variance stabilization normalization
#prot.Norm <- wrapper.normalizeD(prot.Filt1, method = "vsn",
#                                 type="overall", conds=legend)
violinPlotD(prot.Norm, legend=legend, conds = legend)
```

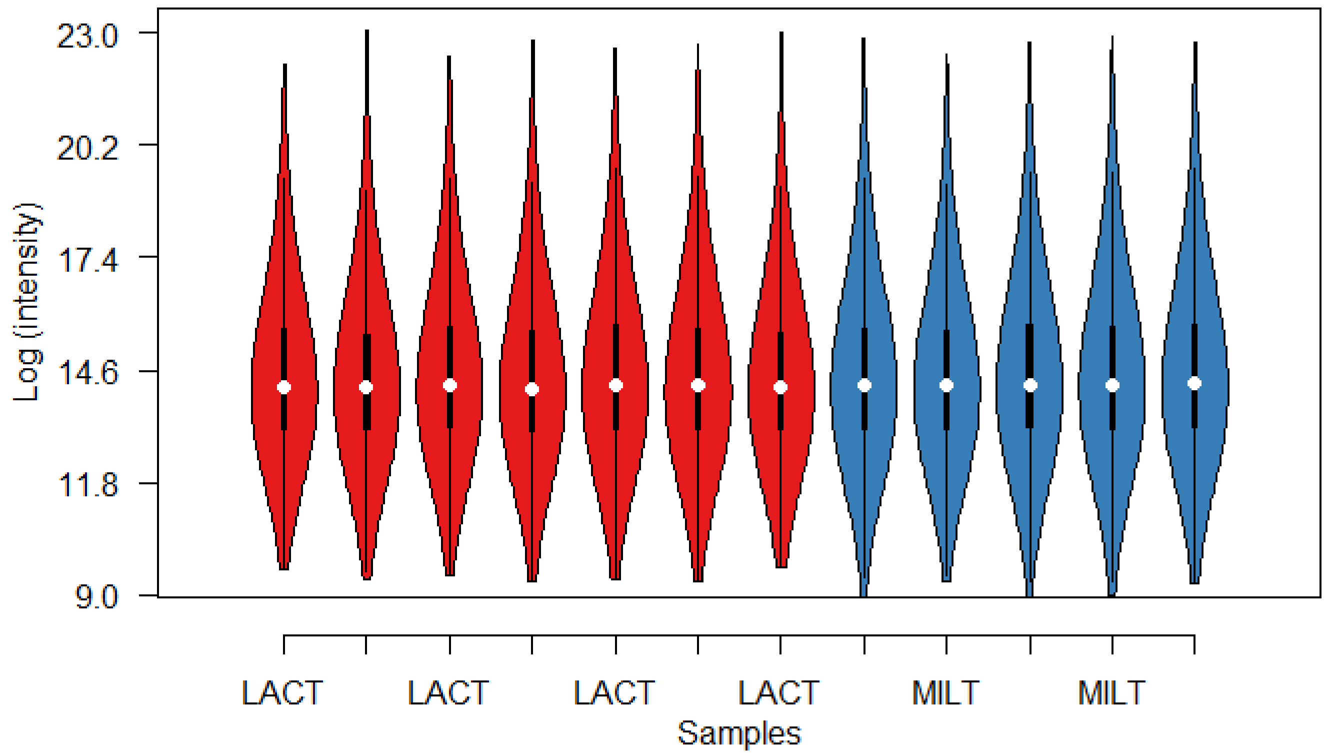

Hide

```
saveRDS(prot.Norm, file="MSnSET-DIA/hSAEC_RSV_ZL_GBUP_NOV2021.norm.msnset")
```

#“Imputation and Missing Values”

Hide

```
#showing number of protein groups missing in an entire condition
metacellHisto_HC(prot.Norm, pattern="missing MEC", indLegend="auto", showValues=TRUE)
```

```
Warning in metacellHisto_HC(prot.Norm, pattern = "missing MEC", indLegend = "auto",  :
  Color palette set to default.
```

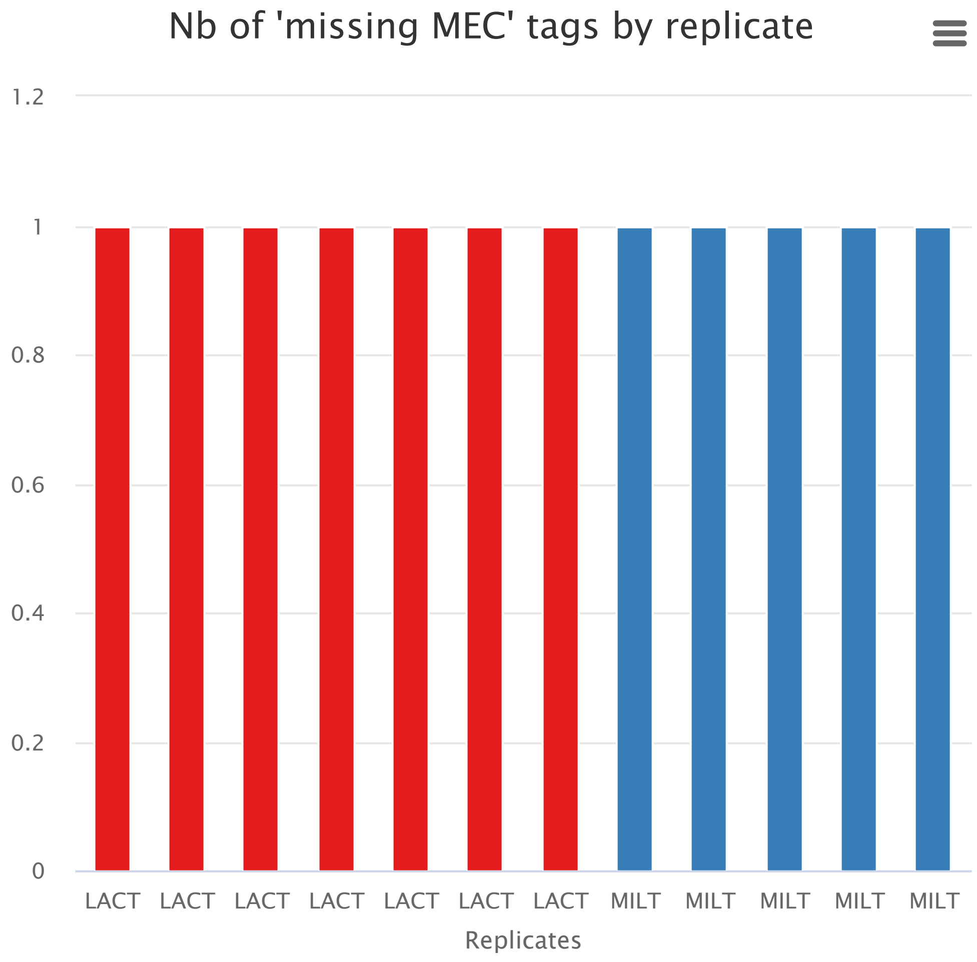

Hide

```
#showing partially observed values missing in a replicate
metacellHisto_HC(prot.Norm, pattern="missing POV", indLegend="auto", showValues=TRUE)
```

```
Warning in metacellHisto_HC(prot.Norm, pattern = "missing POV", indLegend = "auto",  :
  Color palette set to default.
```

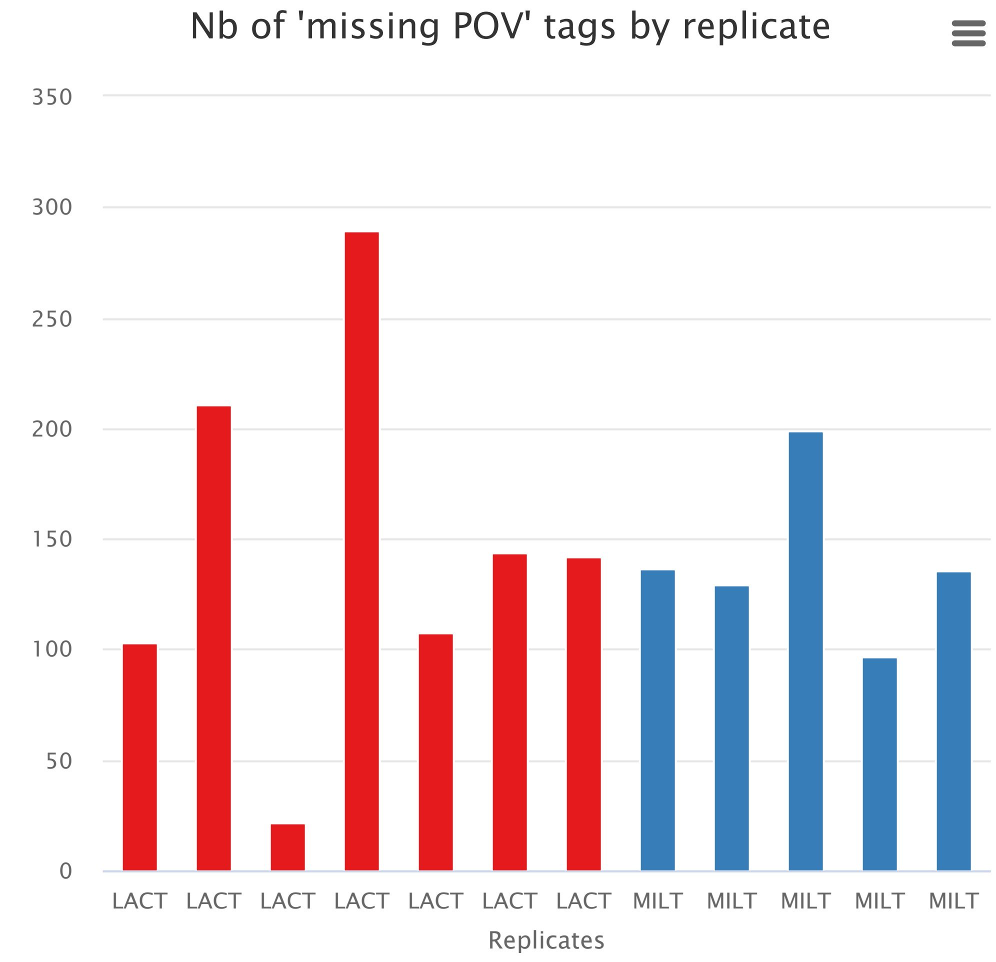

Hide

```
#showing missing value heat map
mvImage(qData, labels)
```

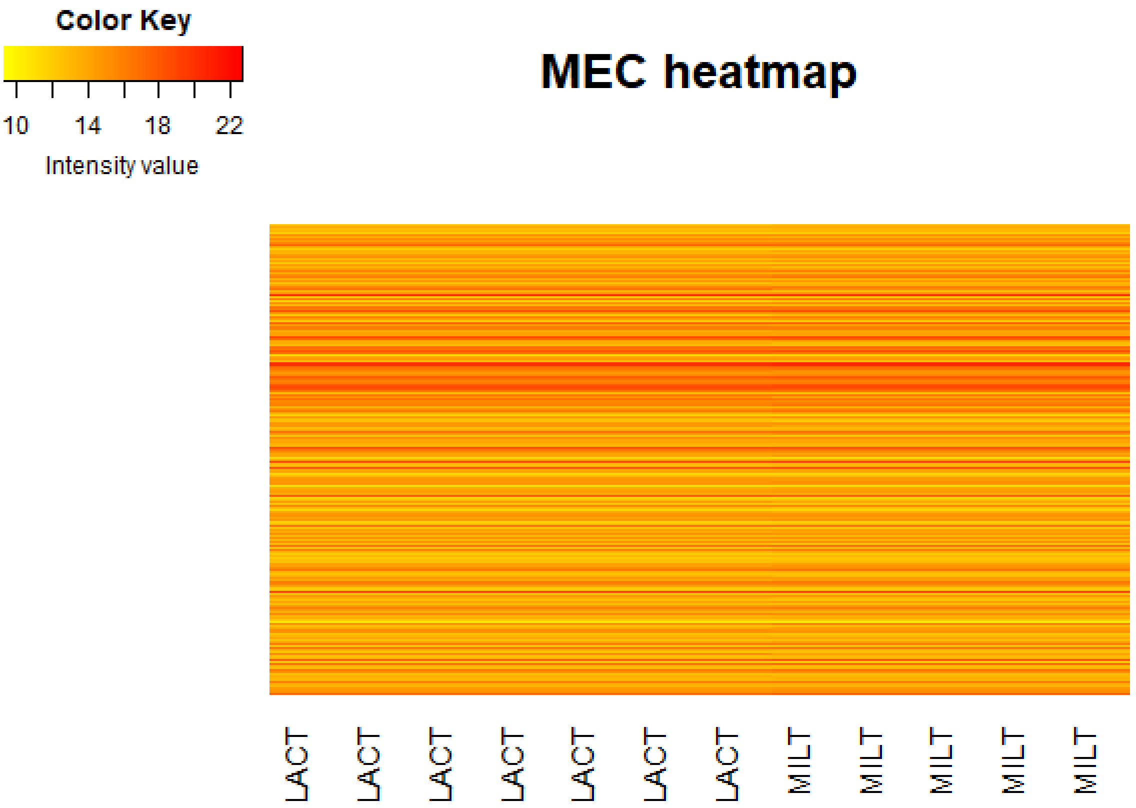

#creates publication quality figure from the missing value matrix

Hide

```
mv_heatmap <- Heatmap(mv_matrix,
        column_split = pData_mv$Condition,
        column_order = pData_mv$Sample.name,
        show_row_names = FALSE,
        show_column_names = FALSE,
        show_column_dend = FALSE,
        show_row_dend = FALSE,
        raster_by_magick = TRUE,
        col=col.fun,
        show_heatmap_legend = FALSE
        ); draw(mv_heatmap)
```

```
`use_raster` is automatically set to TRUE for a matrix with more than 2000 rows. You can control
`use_raster` argument by explicitly setting TRUE/FALSE to it.

Set `ht_opt$message = FALSE` to turn off this message.
```

#Intensity Heatmaps

Hide

```
pdf("DIA-MS L2FC Matrix.pdf", width=5, height=4); draw(mv_heatmap); while (!is.null(dev.list()))
dev.off()
```

```
Error in h(simpleError(msg, call)) :
  error in evaluating the argument 'object' in selecting a method for function 'draw': object 'm
v_heatmap' not found
```

#Summary Statistics

#Differential Abundance Analysis (switching packages from DAPAR)

Hide

```
#quantative data
qData <- Biobase::exprs(prot.Imp2)
#undo log2 to not do so twice
prot.quant <- as.data.frame(2^(qData)); prot.quant$Protein.Group <- rownames(prot.quant)

index <- exData[c(1:4)]
prot.gene <- merge(prot.quant, index, by="Protein.Group")

library(DEP)
```

```
Attaching package: 'DEP'

The following object is masked from 'package:MSnbase':

    impute

The following object is masked from 'package:ProtGenerics':

    impute
```

Hide

```
library(tidyverse)
#make neat list of protein for DEP
prot.unique <- make_unique(prot.gene, "Genes", "Protein.Group",
                           delim=";")

pData <- Biobase::pData(prot.Imp2); #eD <- pData %>% separate(Condition, c("group", "conditio
n"), sep=2) ; eD$replicate <- rep(1:3)
#colnames(eD) <- c("label", "condition", "group", "replicate")

eD <- pData %>% mutate(replicate = str_sub(Sample.name, -1, -1))
colnames(eD) <- c("label", "condition", "replicate")


prot_se <- make_se(prot.unique, c(2:13), eD)
#Below is needed if you have several sample comparisons
#contrasts <- c("NDV_vs_NDC", "ZLV_vs_ZLC", "ZLV_vs_NDC", "ZLC_vs_NDC", "ZLV_vs_NDV")
prot.dif <- test_diff(prot_se, type = "all")
```

```
Tested contrasts: LACT_vs_MILT
```

Hide

```
#prot.dif <- test_diff(prot_se, type = "manual", test = contrasts)
prot.sig <- add_rejections(prot.dif, alpha=0.05, lfc=1)
#have to pull specific columns below
data_results.0 <- get_results(prot.sig)[-c(2,5,6,8,9)]
#data_results <- get_results(prot.sig)[c(1:20, 22:27)]

index2 <- prot.unique %>%
  select(name, Protein.Group, Protein.Names, Genes)

data_results <- merge(data_results.0, index2, by="name") %>%
  select(-name) %>% relocate(where(is.character))
```

#alternative to BH adjustment

Hide

```
library(IHW)
```

```
Attaching package: ‘IHW’

The following object is masked from ‘package:ggplot2’:

    alpha
```

Hide

```
library(tcltk)
library(tidyverse)
library(reshape2)
```

```
Attaching package: ‘reshape2’

The following object is masked from ‘package:tidyr’:

    smiths
```

Hide

```
precFile <- tk_choose.files()
precData <- read.table(precFile, sep="\t", header=TRUE, quote="")

precIndex <- c(11:22)

Condition <- eD$condition
#Condition <- rep(c("NDC", "NDV", "ZLC", "ZLV"), each=3)
#Sample.name <- paste0(Condition, "_", seq(1:3))
Sample.name <- eD$label

colnames(precData)[precIndex] <- Sample.name

precData.summary <- precData %>%
  filter(Protein.Group!="") %>%
  group_by(Protein.Group) %>%
  summarise(number = length(unique(Stripped.Sequence)))

data_reshape <- reshape2::melt(merge(data_results, precData.summary, by="Protein.Group"),
                               id.vars = c("Protein.Group", "Protein.Names", "Genes", "numbe
r"))%>%
  separate(variable, c("contrast", "statistic"), sep=12) %>%
  pivot_wider(id_cols = c("Protein.Group", "Protein.Names", "Genes", "number", "contrast"), name
s_from = "statistic", values_from = "value")
  #dcast(Protein.Group + Protein.Names + Genes + contrast + number~ statistic)

colnames(data_reshape)[c(6:8)] <- c("p.val", "p.adj", "ratio")

p.adj_ihw <- ihw(p.val ~ number, data=data_reshape, alpha=.05)
data_reshape$p.adj <- adj_pvalues(p.adj_ihw)
#data_reshape$contrast <- factor(data_reshape$contrast,
#                                levels = paste0(contrasts,"_"),
#                                #labels = c("condition")
```

#you have the heart of your analysis (data reshape file)

Hide

```
data_reshape[data_reshape$Genes %nin% protList,]$label <- NA
```

```
Error in h(simpleError(msg, call)) :
  error in evaluating the argument 'table' in selecting a method for function '%in%': object 'pr
otList' not found
```

Hide

```
library(tidyverse)
qData <- Biobase::exprs(prot.Imp2)
colnames(qData) <- metaData$Sample.name

#protList <- c("BRD4", "RELA", "JUN", "JUNB", "CTNNB1", "JUP", "JUND", "FOS", "FOSL1", "FOSL2")
protList <- c("TTN", "TNNI3")
#protList <- c("HNRNPD", "SRSF1", "SRSF9", "SNRPD1", "SNRPE", "U2AF2", "PPIH", "PPIL3", "PPIL4", "POLR2G", "DNAJC8", "ELAVL1",
#              "GTF2F2", "CPSF3")
#protList <- c("AKAP17A", "HNRNPD", "SRSF1", "SRSF9", "SNRPD1", "SNRPE", "U2AF2", "PPIH", "PPIL3", "PPIL4", "POLR2G", "DNAJC8")
#protList <- c("F", "P", "G", "1B", "1C", "L")
#protList <- c("GFPT1", "GFPT2", "GNPNAT1", "PGM3", "UAP1",
#              "ALG1", "ALG2", "ALG5", "ALG9", "ALG11",
#              "STT3A", "STT3B")
#protList <- c("IFRD1", "EIF4A2")
index <- exData[c(2:4)]
protList.df <- index[index$Genes %in% protList,]
qData.filt <- as.data.frame(qData[rownames(qData) %in% protList.df$Protein.Ids,]); qData.filt$Protein.Ids <- rownames(qData.filt)

protList.qD <- merge(qData.filt, index, by="Protein.Ids") %>%
  pivot_longer(cols=2:13) %>% separate(name, c("group", "replicate"), sep="_")
protList.qD$Genes <- factor(protList.qD$Genes,
                            levels=protList)

library(ggpubr)
boxplots <- ggboxplot(protList.qD, x="group", y="value", add="jitter") +
  facet_wrap(~Genes, scales="free") +
  scale_x_discrete(name=element_blank()) +
  scale_y_continuous(name=expression(Log[2]*" LFQ Intensity"),
                     breaks=scales::pretty_breaks(n=5),
                     expand=expansion(mult=c(.1,.5))) +
  theme(strip.background = element_blank(),
        strip.text = element_text(family="sans",
                                  face="plain",
                                  size=14),
        panel.border = element_rect(color="black", fill=NA)); print(boxplots)
```

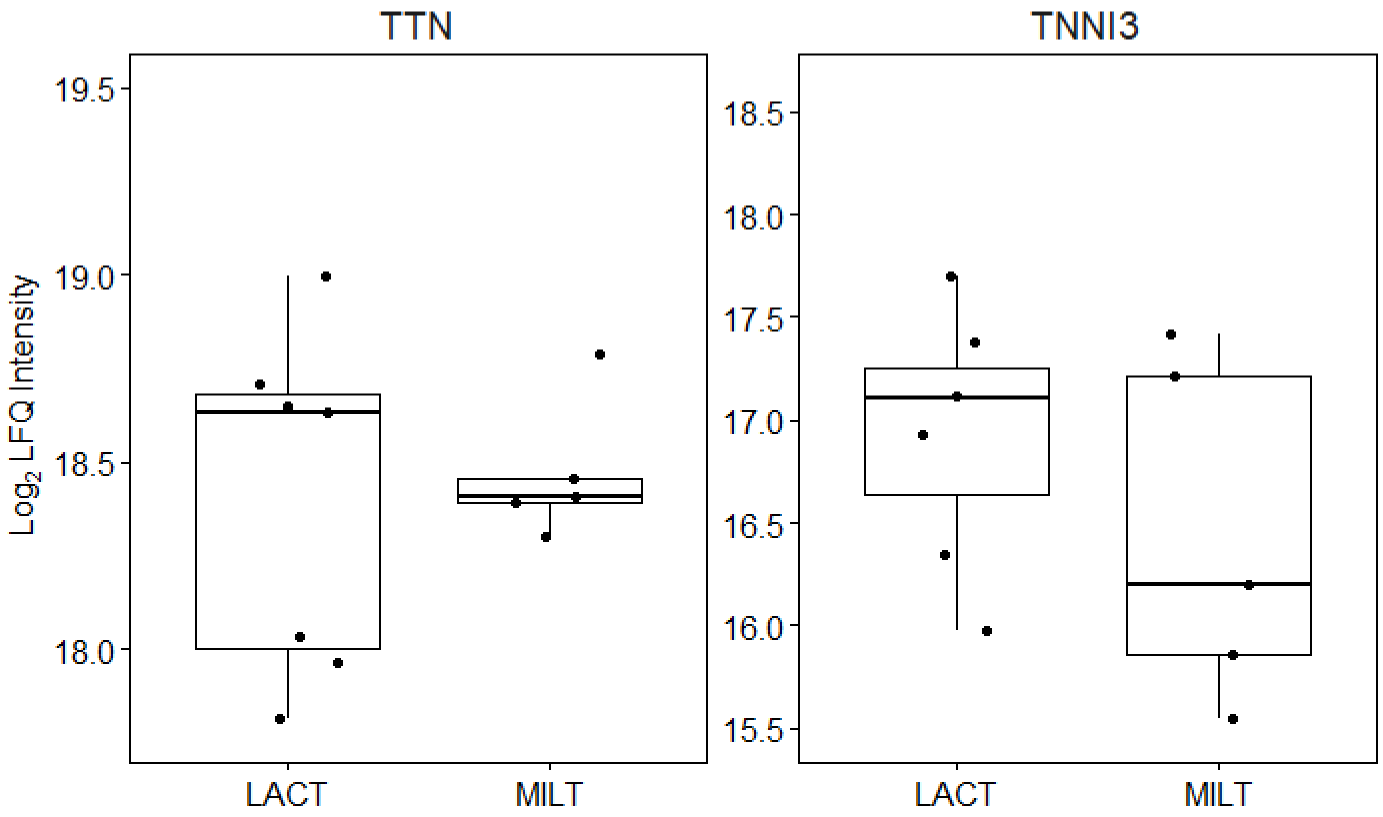

Hide

```
#ggsave("Plots/DIA-Example Boxplot.png", width=5, height=2, units="in")
```

Hide

```
library(DAPAR)
expr <- t(Biobase::exprs(prot.Imp2))

library(ggfortify)
library(RColorBrewer)
library(ggpubr)
expr.pca <- prcomp(expr)
df_out <- as.data.frame(expr.pca$x)
df_out$Condition <- metaData$Condition

percentage <- round(expr.pca$sdev^2 / sum(expr.pca$sdev^2) * 100,2)
percentage <- paste( colnames(df_out), "(", paste( as.character(percentage), "%", ")", sep="") )

pca.plot <- ggplot(df_out, aes(x=PC1, y=PC2, color=Condition)) +
  geom_point(size=2.5, alpha=0.5) +
  scale_color_manual(values=brewer.pal(6, "Dark2")) +
  xlab(percentage[1]) + ylab(percentage[2]) +
  theme_bw() +
  theme(legend.position = c(.85,.75),
        axis.text = element_text(
          size=12),
        axis.title = element_text(
          size=14),
        panel.border = element_rect(size=1),
        axis.ticks = element_line(
          color="black", size=1
        )); print(pca.plot)

ggsave("DIA-PCA.png", width=6, height=5, units="in")
```

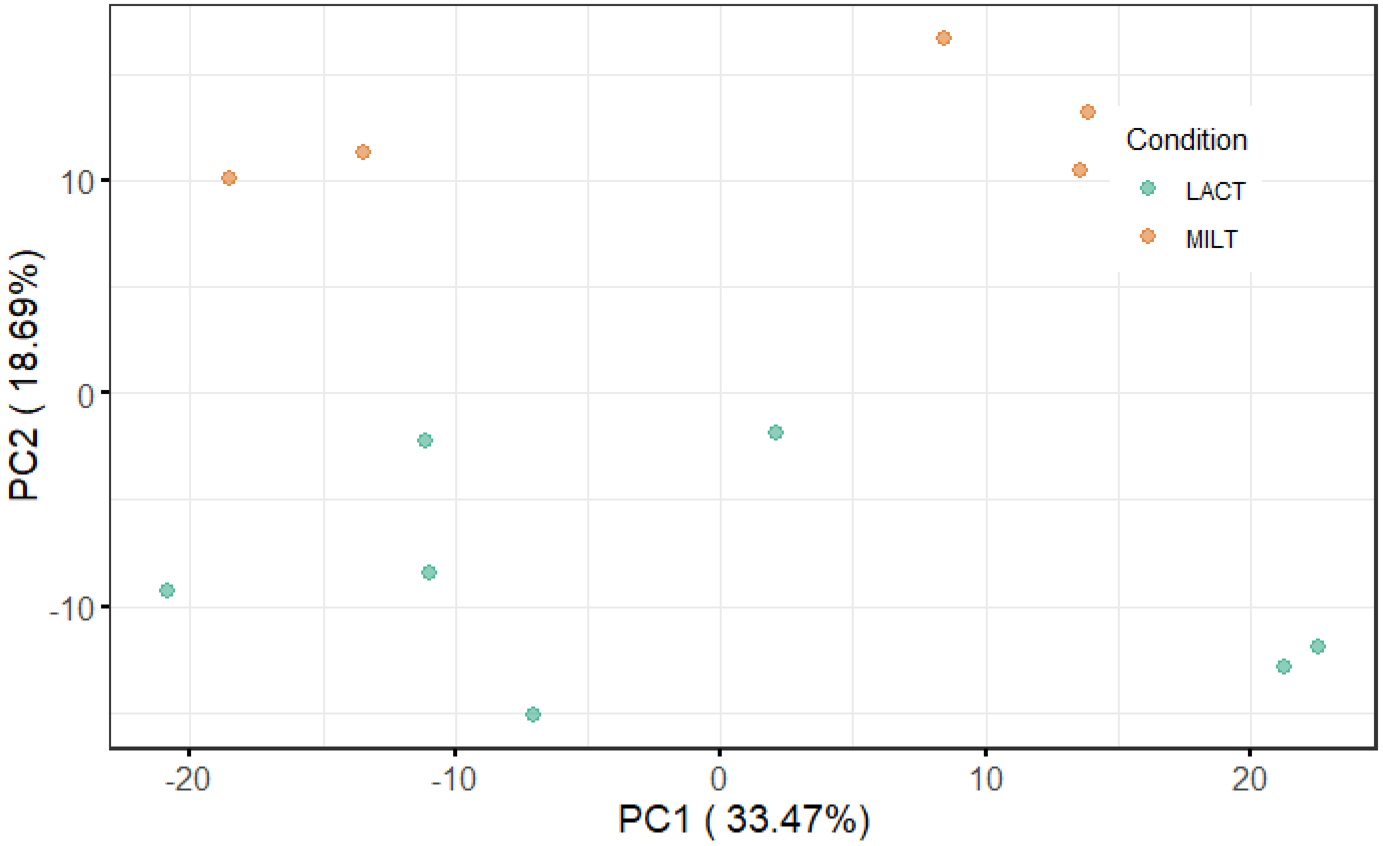

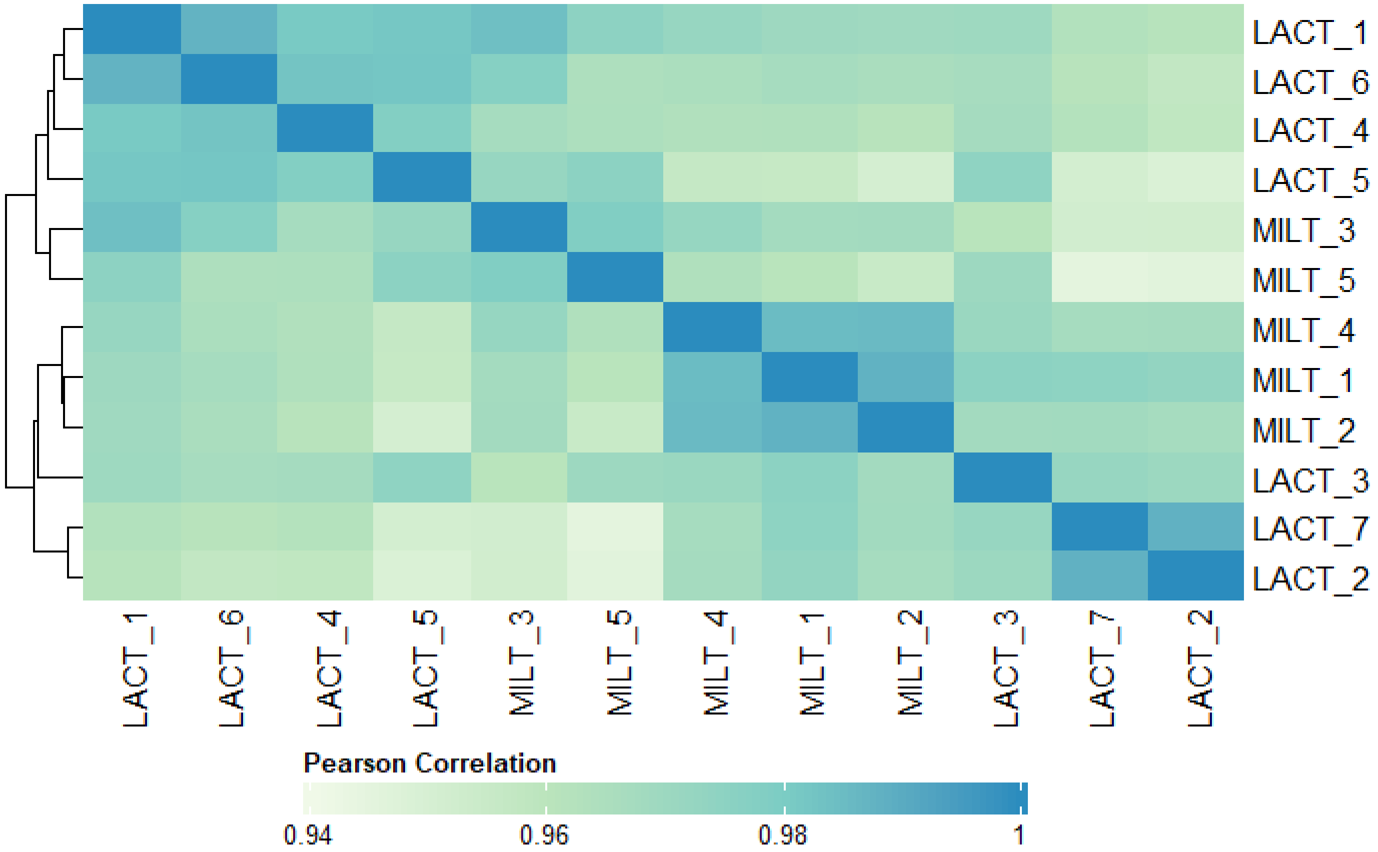

#Reactome Pathways

Hide

```
react.path <- read.delim(react.file, header = FALSE, sep="\t",
                         col.names=c("UNIPROT", "reactomeID", "URL", "pathwayName", "evidenceCod
e", "species")) %>%
  filter(species == "Homo sapiens") %>%
  dplyr::select(-c(URL, evidenceeCode, species))
```

```
Error in `dplyr::select()`:
! Can't subset columns that don't exist.
x Column `evidenceeCode` doesn't exist.
Backtrace:
  1. ... %>% dplyr::select(-c(URL, evidenceeCode, species))
  3. dplyr:::select.data.frame(., -c(URL, evidenceeCode, species))
  6. tidyselect::eval_select(expr(c(...)), .data)
  7. tidyselect:::eval_select_impl(...)
 16. tidyselect:::vars_select_eval(...)
     ...
 22. tidyselect:::reduce_sels(node, data_mask, context_mask, init = init)
 23. tidyselect:::walk_data_tree(new, data_mask, context_mask)
 24. tidyselect:::as_indices_sel_impl(...)
 25. tidyselect:::as_indices_impl(x, vars, call = call, strict = strict)
 26. tidyselect:::chr_as_locations(x, vars, call = call)
```

#Pathway violin plots

Hide

```
l2fc.boxplot <- ggplot(
  df2, aes(x=contrast, y=ratio, label=label)
) +
  geom_boxplot(color="black") +
  geom_point(position = "jitter") +
  geom_text_repel() +
  facet_wrap(~pathwayName) +
  scale_x_discrete(name=element_blank()) +
  scale_y_continuous(name=expression(Log[2]*" Fold Change"),
                     breaks=c(-1.5, -1, -.5, 0, .5, 1, 1.5)) +
  theme_classic(); print(l2fc.boxplot)
```

```
Warning: Removed 260 rows containing missing values
(geom_text_repel).
```

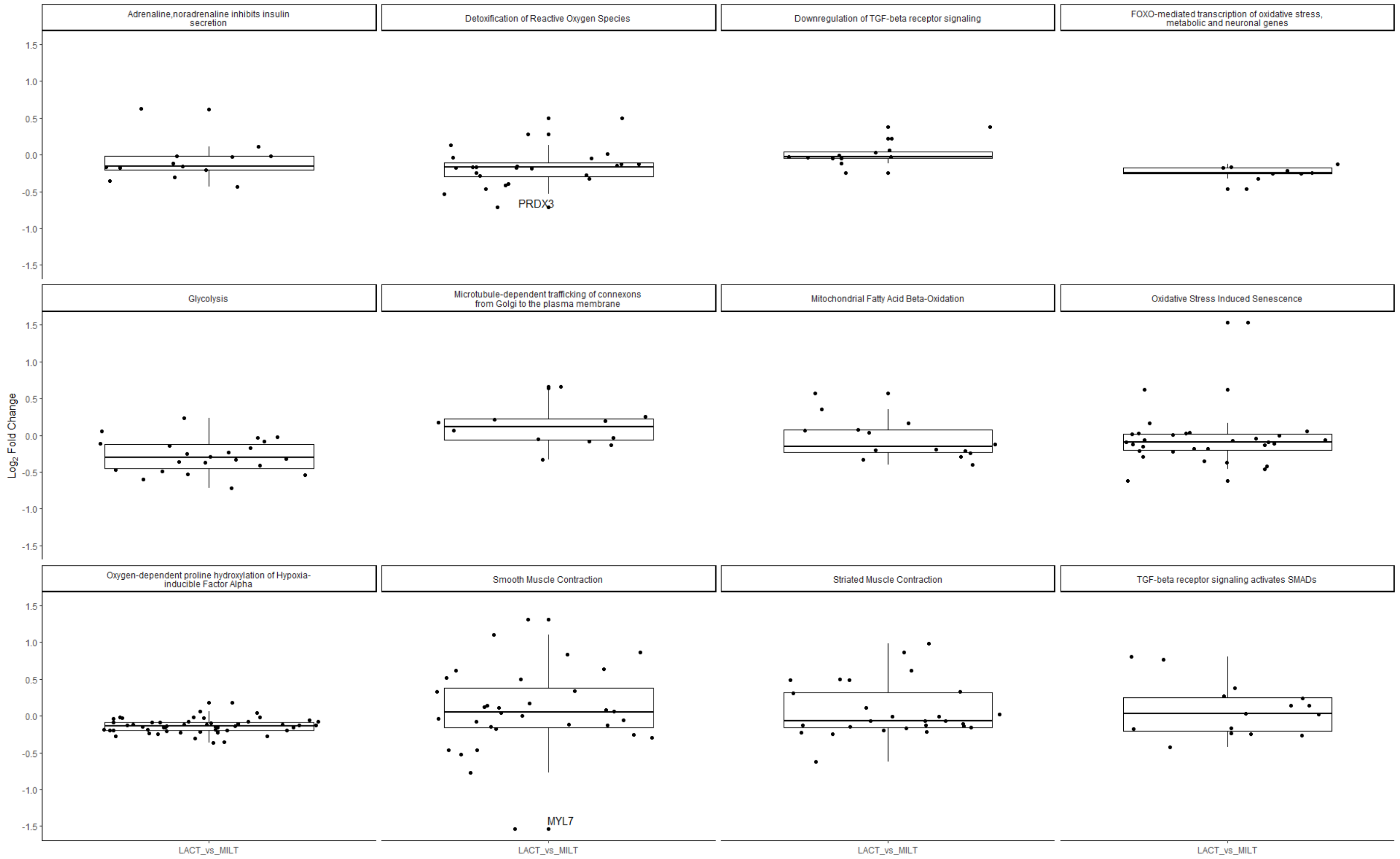

Hide

```
library(tcltk)
library(tidyverse)
library(stringr)
PANTHER_GO <- tk_choose.files()
PANTHER.df <- read.table(PANTHER_GO,
                          sep="\t", header=TRUE)
colnames(PANTHER.df) <- c("process","ref_count","input_count",
                          "expected_count","over_under",
                          "fold_enrichment", "pval", "FDR")

PANTHER.df2 <- PANTHER.df %>%
  mutate(ratio = input_count/ref_count) %>%
  arrange(desc(ratio)) %>%
  mutate(process = substr(process, 1, nchar(process)-13)) %>%
  mutate(process = str_wrap(process, width=35)) %>%
  filter(over_under=="+") %>%
  slice_head(n=15)
```

```
Error in `mutate()`:
! Can't transform a data frame with duplicate names.
Backtrace:
 1. ... %>% slice_head(n = 15)
 8. dplyr:::mutate.data.frame(., ratio = input_count / ref_count)
```

#export files to excel

Hide

```
install.packages("writexl")
```

```
Warning in install.packages :
  the 'wininet' method is deprecated for http:// and https:// URLs
```

```
trying URL 'https://cran.rstudio.com/bin/windows/contrib/4.2/writexl_1.4.0.zip'
Content type 'application/zip' length 190346 bytes (185 KB)
downloaded 185 KB
```

```
package ‘writexl’ successfully unpacked and MD5 sums checked

The downloaded binary packages are in
    C:\Users\kjreese\AppData\Local\Temp\1\RtmpqKktLP\downloaded_packages
```
